# Pd-Coordinated Salinidol-Modified Mixed MOF: An Excellent Active Center for Efficient Nitroarenes Reduction and Selective Oxidation of Alcohols

**DOI:** 10.1021/acsomega.3c02414

**Published:** 2023-06-07

**Authors:** Hassan Keypour, Jamal Kouhdareh, Sedigheh Alavinia, Rahman Karimi-Nami, İdris Karakaya

**Affiliations:** † Faculty of Chemistry, Bu-Ali Sina University, Hamedan 65174, Iran; ‡ Department of Organic Chemistry, Faculty of Chemistry, Bu-Ali Sina University, Hamedan 65174, Iran; § Department of Chemistry, Faculty of Science, University of Maragheh, Maragheh 55181-83111, Iran; ∥ Department of Chemistry, College of Basic Sciences, Gebze Technical University, Gebze 41400, Turkey

## Abstract

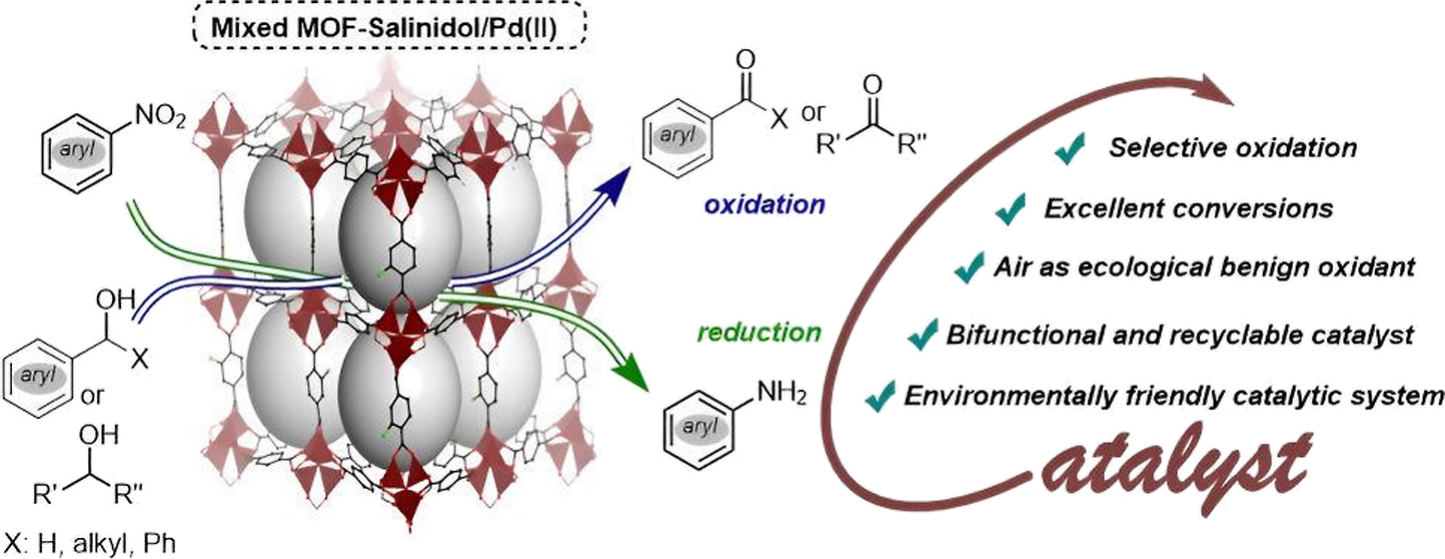

Selective oxidation of active and inactive alcohol substrates and reduction of nitroarenes is a highly versatile conversion that remains a challenge in controlling functionality and adjustments in metal–organic frameworks (MOFs). On the other hand, it offers an attractive opportunity to expand their applications in designing the next generation of catalysts with improved performance. Herein, a novel mixed MOF consisting of supported 2-hydroxybenzamide (mixed MOF-salinidol) has been fabricated by post-synthetic modifications of mixed MOF. Subsequently, the prepared nanocomposites were modified to impart catalytic sites using palladium chloride ions mixed with MOF-salinidol/Pd (II). After successfully designing and structurally characterizing nanocomposites, we evaluated their activity in oxidizing primary and secondary alcohols using aerobic conditions with molecular oxygen and an air atmosphere. In addition, the stability of (mixed MOF-salinidol/Pd (II)) catalysts under catalytic conditions was also demonstrated by comparing the Fourier-transform infrared spectrum, scanning electron microscopy image, and ICP-OES method before and after catalysis. Based on the results, the active surface area of the synthesized nanocatalyst is large, which highlights its unique synergistic effect between post-synthetic modified MOF and Pd, and furthermore, the availability of catalytic sites from Pd, as demonstrated by outstanding catalytic activity.

## 1 Introduction

Numerous studies have been done since metal–organic frameworks (MOFs) appeared in our world nearly two decades ago, and new studies continue to be done with increasing interest. Properties such as having large surface areas, a highly porous structure, and easy functionalization can be counted among the important features of MOFs that deserve this attention. They have many cutting-edge applications like storing gas,^1,2^ catalyzing processes,^3,4^ delivering drugs,^5−7^ encapsulating material,^8^ supercapacitors,^9−12^ absorbent of heavy metals,^3,13^ and other uses. MOFs not only have a higher activation and durability level than other classes of porous substances but also can change the morphology and size of cavities. This has become a benefit in respect of differentiation and greater selectivity in their uses.^14^

Post-synthetic modification of MOF is a versatile method for generating catalysts with advantageous multiple active sites, which is why this type of material is becoming well received by researchers.^15^ Creating novel molecular scaffolds with distinct structural and biological features to enhance their capacity and selectivity presents an intriguing task.^16^ The use of mixed organic ligands in the synthesis of MOFs has attracted the attention of researchers in recent years. By mixing these ligands, synthesized MOFs with different properties are obtained from the species synthesized with a single ligand.^17^

MOFs among the nanoporous compounds are stable under various conditions and can maintain porosity due to their chemical and thermal resilience.^18^ In the last few years, there have been multiple reports of the excellent performance of altered catalysts in MOF structures, specifically in oxidation, reduction reactions, carbon–carbon bonding, and more.^19^ In some functional reports, MOFs have been suggested as more popular candidates than other porous nanostructures.^20^ One of the most important challenges in catalytic processes is investigating the applications of post-synthetic MOF processes and the formation of palladacycle complexes for use in oxidation and reduction reactions.^21−26^

Although MOFs have great potential as heterogeneous catalysts and have attracted significant interest from researchers, plans to use them at the industrial stage have yet to make significant progress.^27^ Catalyst encapsulation protection is one of the most important advantages of using MOFs. Confinement of the active species within the pores can provide the catalyst with some degree of protection from other reactive species, but it is difficult to achieve in homogeneous phases through ligand manipulation.^28−31^

A flexible MOF allows you to adjust the breathing action, especially the pressure at which the MOF opens. Heterogeneous catalysis enables new cascade and tandem reactions, with particular attention to reactions in which two metals nearby actually form mixed-metal transition states. Bimetallic MOFs should have distinct advantages over the corresponding mixtures of monometallic MOFs, and bimetallic enzymes may be a great source of inspiration in this field.^32^

Alcohol oxidation reactions play a vital role in industrial applications, the synthesis of synthetic intermediates, and natural products.^33^ Based on this importance, studies involving new catalyst systems, new oxidants, and new methodologies continue to attract attention. Alcohol oxidation is widely carried out traditionally with small organic-based reagents such as Dess-Martin periodinane and Swern or metal-based systems such as pyridinium chlorochromate, pyridinium dichromate, and ruthenium tetroxide.^34−37^ However, most of them have various limitations with at least one property, such as being moisture-sensitive, expensive, and not reusable. Therefore, in new approaches, oxidations are expected to be carried out selectively with green, cheap, and non-toxic oxidants such as dioxygen or air in the presence of reusable catalysts.^38^

Because nitrobenzenes are toxic, they adversely affect humans and other organisms and are also known as environmental pollutants. Therefore, developing new catalytic systems for effective and economical removal methods is important.^39^ Besides, the reduction of nitrobenzenes to aniline derivatives is quietly significant synthon in organic synthesis and is present in a variety of pesticides, pharmaceuticals, and fine chemicals.^40^ However, although heterogeneous catalysts with metals such as Pd,^41^ Au,^42^ Pt,^43^ and Mo^44^ have been developed so far, selective reduction of nitro compounds is still a challenge under mild conditions.

Regarding the mentioned points, the present article introduces the design and characterization of a new Zn-based MOF featuring 2-hydroxybenzoic acid covalently bonded to the IRMOF-3&IR-MOF-1 (mixed MOF) *via* post-synthetic modifications. Palladium (Pd) species were then successfully decorated on prepared support (mixed MOF-salinidol/Pd(II)). The catalytic activities of this successfully synthesized nanocatalyst were tested in aerobic oxidation reactions of alcohols and reduction reactions of nitroarene compounds. In light of the results obtained, the high efficiency and easy recovery of the catalyst are strong points to justify its use.

## 2 Experimental Section

### 2.1 Synthesis of Mixed MOF **3**

**Scheme 1:**
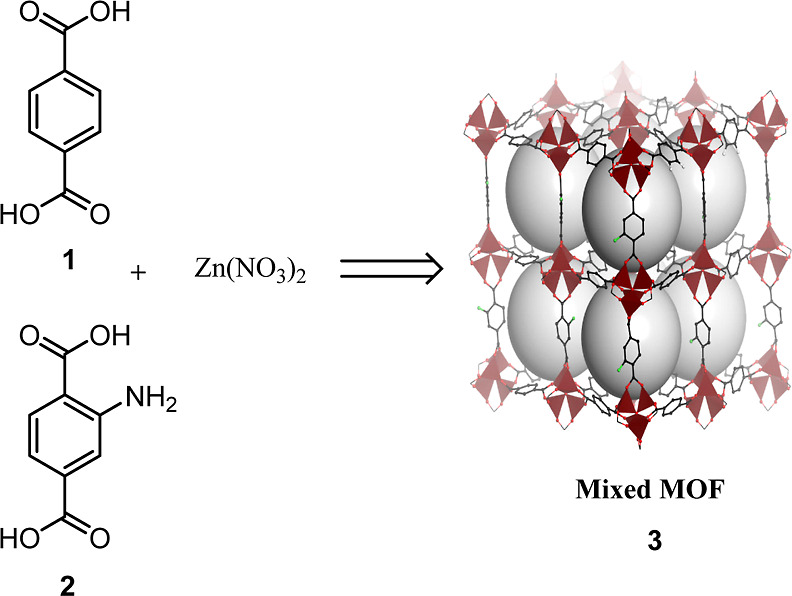
Preparation of Mixed MOF **3**

Zn(NO_3_)_2_·6H_2_O (1.80 g, 6 mmol), 2-aminoterephthalic acid **2** (0.18 g, 1 mmol), and terephthalic acid **1** (0.17 g, 1 mmol) were dissolved in DMF (50 mL) by sonication. The solution was obtained after ensuring the container was closed and impermeable for 1 day in the oven at 100 °C. Next, the obtained crystals were separated, washed with DMF (10 mL) and chloroform (30 mL), and then immersed in chloroform for 1 day to remove DMF guest molecules, and finally dried at 50 °C under reduced pressure^15^ (Scheme 1).

### 2.2 Synthesis of Modified Mixed MOF-Salinidol **6**

**Scheme 2:**
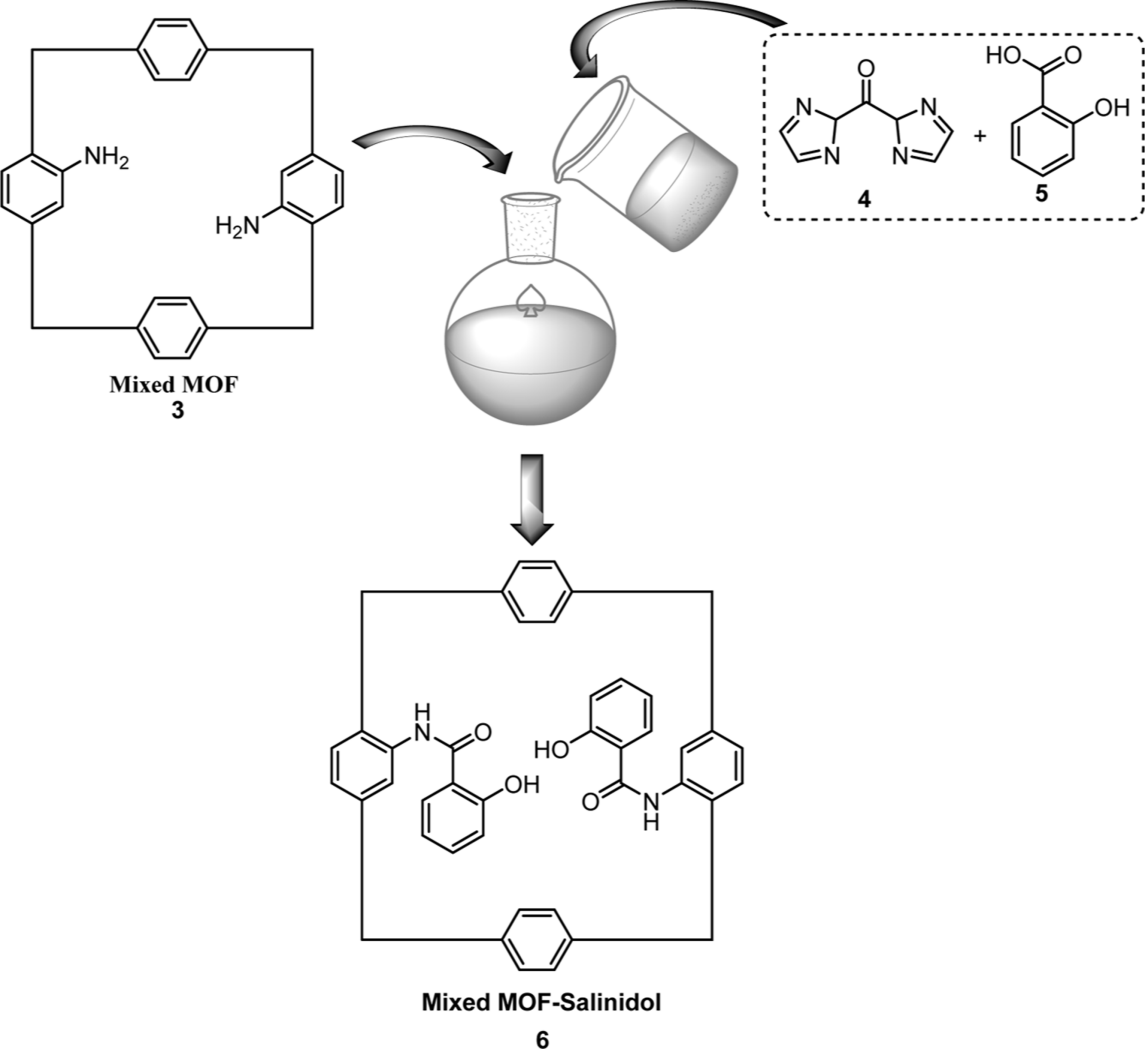
Preparation of Mixed MOF-Salinidol **6**

First, 300 mg of mixed MOF **3** was dissolved in 20 mL of DMF for modified catalyst palladacycle complexes. Next, in a separate container, salicylic acid **5** (138 mg, 1 mmol) and 1,1′- carbonyldiimidazole (CDI) **4** (20 mg, 0.12 mmol) were dissolved in 50 mL of DMF at 40 °C and stirred for 30 min. Finally, the contents of this container were added to the dispersed solution containing the mixed MOF and heated at 40 °C for 12 h. After filtering, the remaining particles were picked up, immersed in chloroform for 1 day to remove DMF guest molecules from the modified mixed MOF, and dried at 50 °C under reduced pressure (Scheme 2).

**Scheme 3:**
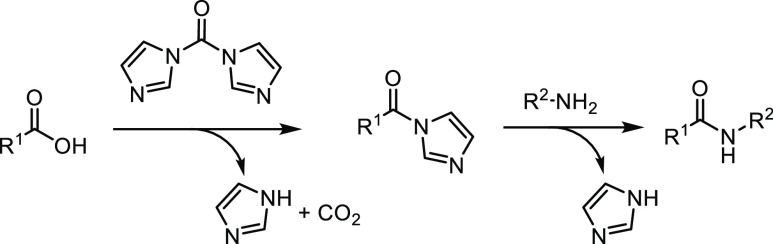
Reaction of Imide Formation Using CDI

The reaction of imide formation using CDI and the carboxylic agent is as follows^45^ (Scheme 3).

### 2.3 Synthesis of Modified Mixed MOF-Salinidol/Pd(II) **7**

**Scheme 4:**
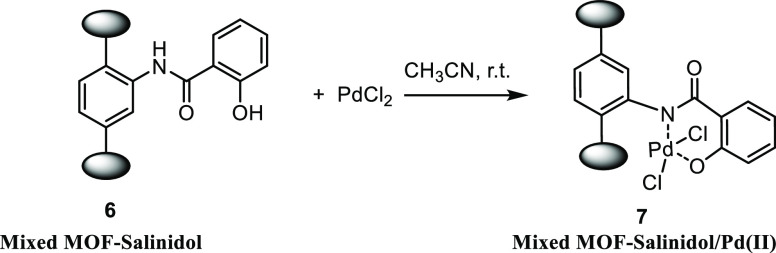
Synthesize of Mixed MOF-Salinidol/Pd(II) **7**

Mixed MOF-salinidol **6** (250 mg) was sonicated in acetonitrile (30 mL) for 30 min. Then, a solution of PdCl_2_ (30 mg) in 20 mL of acetonitrile was added to the dispersed modified mixed MOF acid and stirred for 24 h at ambient temperature. Finally, modified mixed MOF was separated by decantation and washed with acetonitrile (Scheme 4). According to the ICP analysis, the Pd content was 10.6%.

### 2.4 General Procedure for Nitroarene Reduction Using Mixed MOF-Salinidol/Pd(II)

A mixture of H_2_O (10 mL), H_2_ (1 atm), nitroarenes (1.0 mmol), and mixed MOF-salinidol/Pd(II) **7** (100 mg, 0.1 mol %) were added into a reaction flask for 1 h and stirred at 75 °C. Reaction progress was monitored by the TLC. Gas chromatography (GC) was used to determine the reaction conversion.

### 2.5 General Procedure for Benzyl Alcohol Oxidation by Using Mixed MOF-Salinidol/Pd(II)

To a solution of mixed MOF-salinidol/Pd(II) **7** (300 mg, 0.3 mol %) in dry trifluorotoluene (3 mL) was successively added alcohol (1.0 mmol) *via* syringe. The mixture was stirred at 95 °C for 1 h. The reaction mixture was dried (MgSO_4_) and concentrated in a vacuum. The conversion was determined by GC.

## 3 Results and Discussion

### 3.1 Catalyst Characterization

**Figure 1:**
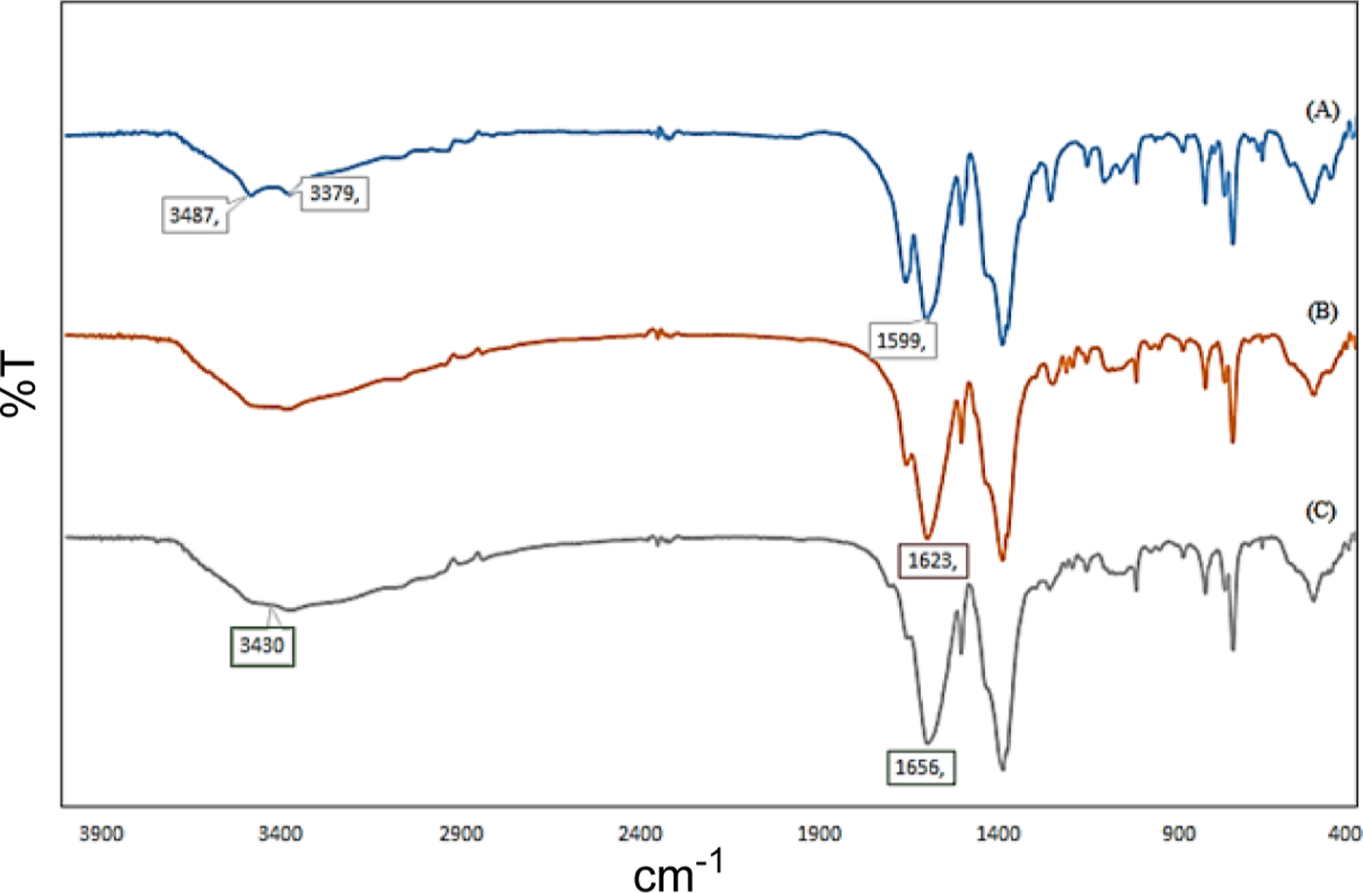
FT-IR spectra of (A) mixed MOF **3**, (B) mixed MOF-salinidol **6**, and (C) mixed MOF-salinidol/Pd (II) **7**.

Figure 1 shows the Fourier-transform infrared spectroscopy (FT-IR) absorption spectra of mixed MOF **3**, mixed MOF-salinidol **6**, and mixed MOF-salinidol/Pd(II) **7**. The stretching vibrations at 3400 cm^–1^ in the FT-IR spectrum of mixed MOF **3** indicate that the symmetric modes of the N–H bonds are not attached to the Zn atoms (Figure 1A). Comparing the spectra of mixed MOF-salinidol **6**, and mixed MOF-salinidol/Pd(II) **7** (Figure 1B,C with Figure 1A) confirmed the successful functionalization. The addition of Pd species to the structure of mixed MOF-salinidol **6** significantly changes the FT-IR spectra (1685 vs 1665 cm ^–1^) (Figure 1B,C).

**Figure 2:**
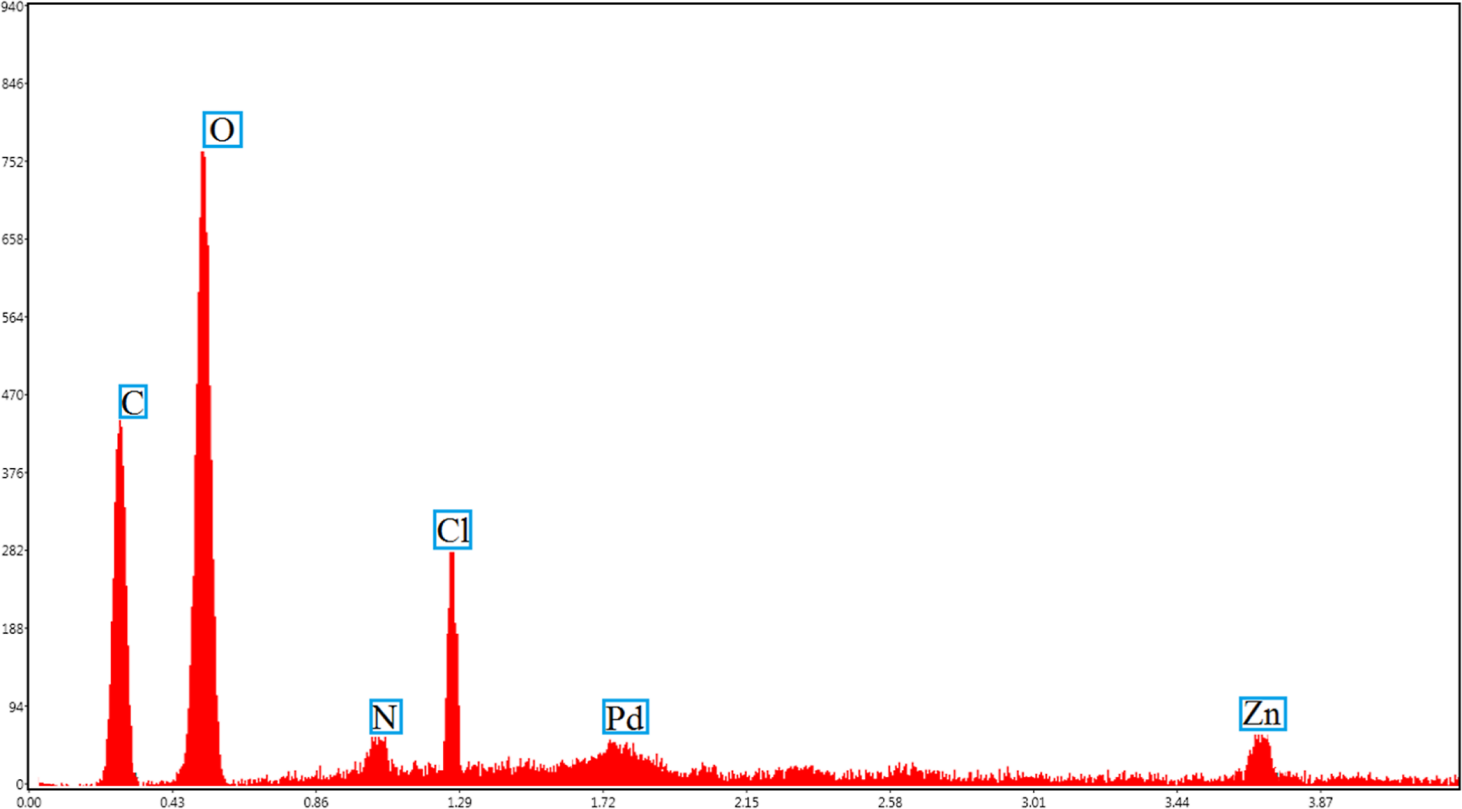
EDX analysis of the mixed MOF-salinidol/Pd(II) **7**.

The energy-dispersive X-ray (EDX) spectroscopy result proves the presence of all expected elements in the structure, such as carbon, nitrogen, oxygen, Pd, and zinc (Figure 2). Furthermore, scanning electron microscopy (SEM)-coupled EDX data for the synthesized nanocatalyst show that it contains C (38.0), O (47.2), N (5.45), Cl (1.47), Pd (2.99), and Zn (4.89).

**Figure 3:**
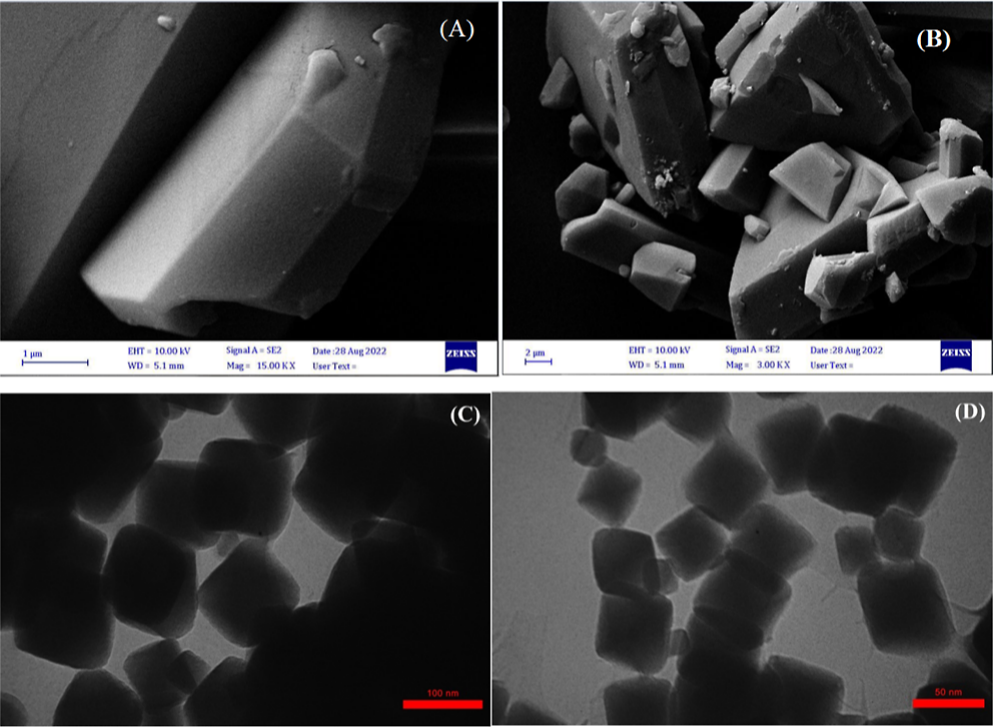
SEM images of mixed MOF-salinidol/Pd(II) (A,B) **7**, TEM images of mixed MOF-salinidol/Pd(II) (C,D) **7**.

SEM and transmission electron microscopy (TEM) analysis were applied to examine the morphological and chemical changes of mixed MOF-salinidol/Pd(II) **7**. The SEM image of the mixed MOF-salinidol/Pd (II) **7** catalyst indicates almost cubic structure morphology, showing no significant change even after the immobilization of metal species. However, it should be noted that mixed MOF can retain Pd species in the pores and prevent their agglomeration (Figure 3A,B). In addition, the TEM images of mixed MOF-salinidol/Pd(II) 7 showed that the mixed MOF particles were bulk cubic, and the size of cubic particles was found to be in the range of ∼50–100 nm. In MOFs, due to the attachment of Pd particles inside the cavities, TEM images may not clearly show the presence of these particles in the inner layers according to the identification mechanism of the TEM technique. However, the catalyst appears to contain a large number of small Pd particles with sizes smaller than 1–2 nm (Figure 3C,D).

**Figure 4:**
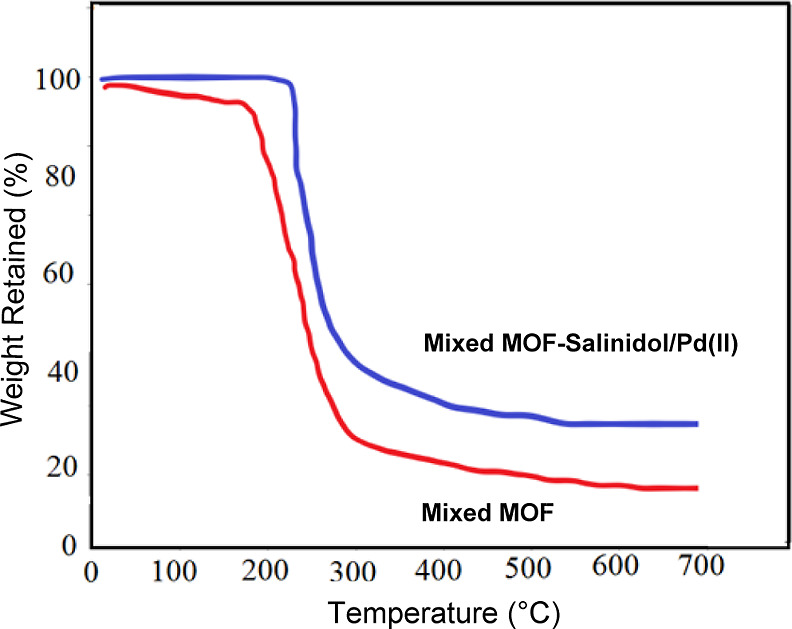
TGA curves of (A) mixed MOF and (B) mixed MOF-salinidol/Pd(II) **7**.

Figure 4 shows the thermogravimetric analysis (TGA) curves showing the residual masses of the samples in the temperature range of 25–700 °C and at a heating rate of 10 °C min^–1^. The TGA curve of pure mixed MOF (Figure 4A) initially displays an imperceptible weight loss (11%) in the region 90–180 °C, which verifies the loss of solvent absorbed at the surface of mixed MOF. A significant weight loss of 71% in the 300–700 °C range can be explained by the decomposition of mixed MOF. The total weight loss is about 82%, again indicating that the mixed MOF decomposes. Figure 4B, depicting the curve for mixed MOF-salinidol/Pd(II) **7**, is similar to Figure 4A and represents a minor weight loss in the range of 30–200 °C, presumably because of the evaporation of the adsorbed solvent. Mixed MOF-salinidol/Pd(II) **7** showed a significant weight loss in the 250–300 °C range, indicating the decomposition of organic matter. It is estimated that 34% of the residual weight is related to Pd and residual zinc. According to Figure 4, the thermal stability of mixed MOF **3** is lower than that of mixed MOF-salinidol/Pd(II) **7**. Hence, it can be concluded that the immobilization of Pd species and organic modification increase the thermal stability of the prepared catalyst.

**Figure 5:**
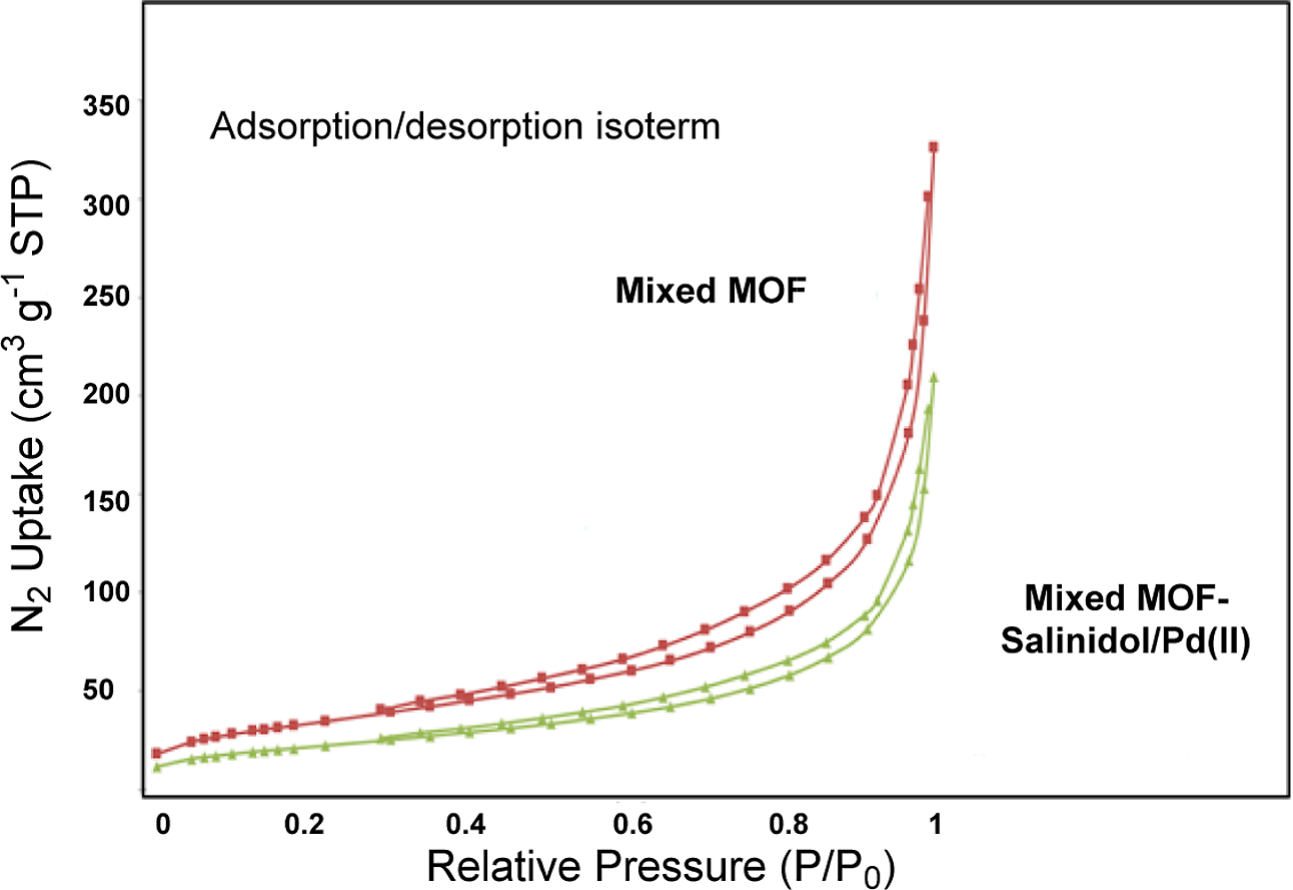
N_2_ adsorption isotherms by the BET analysis of (A) mixed MOF **3** and (B) mixed MOF-salinidol/Pd(II) **7**.

The important parameters associated with the porosity of mixed MOF **3** and mixed MOF-salinidol/Pd(II) **7** samples, including the surface area and total pore volume, and the average diameter of the pores, are reported in Table 1. The total pore volume and surface area of mixed MOF were 545 m^2^ g^–1^ and 0.56 cm^3^ g^–1^, respectively. The catalyst surface area and total pore volume calculated based on the Brunauer–Emmett–Teller (BET) equation were 395 m^2^ g^–1^ and 0.37 cm^3^ g^–1^, respectively. A secondary complex form with Pd(II) has reduced its surface. This surface reduction is normal because secondary complexes of Pd occupy part of the cavity space. Figure 5A,B indicates the N_2_ adsorption isotherms of the samples. The N_2_ adsorption and desorption curves of the samples show type IV isotherms with H_2_ hysteresis curves, which confirm their mesoporous structure.

**Table 1:** Structural Data of the Mixed MOF and Mixed MOF-Salinidol/Pd(II)

sample	surface area (m^2^/g)	mean pore diameter (nm)	total pore volume (cm^3^/g)
mixed MOF **3**	545	0.56	48
mixed MOF-salinidol/Pd(II) **7**	395	0.37	32

**Figure 6:**
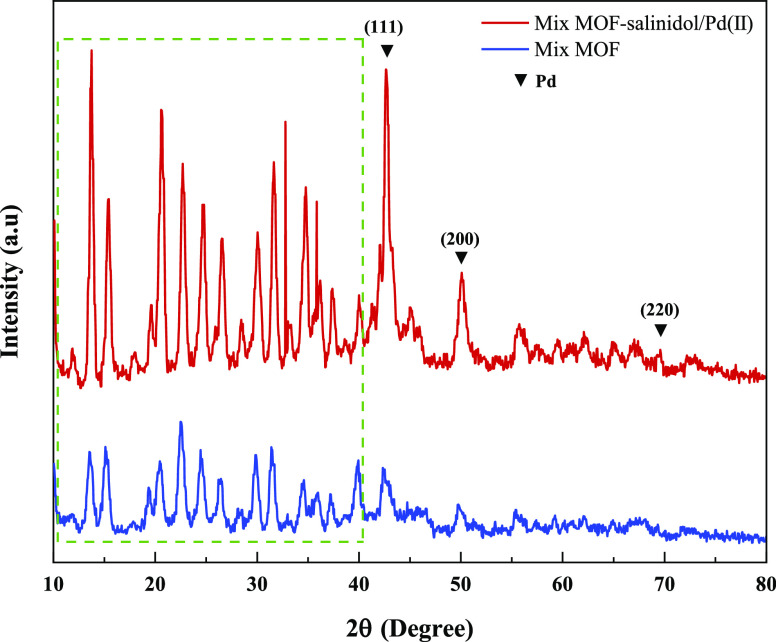
XRD pattern of mixed MOF **3** and mixed MOF-salinidol/Pd(II) **7**.

XRD analysis was used to check the crystal structure of mixed MOF **3** and mixed MOF-salinidol/Pd(II) **7** samples (Figure 6). The major diffraction peaks of mixed MOF agree with the pattern of mixed MOF **3** reported in the literature.^15,46,47^ It can be seen that the XRD pattern of mixed MOF is similar to that of mixed MOF **3** on the main diffraction peaks. In the XRD spectrum of mixed MOF-salinidol/Pd(II) **7**, a minor shift in position and width, as well as the intensity of a few peaks, and the appearance of several sharp peaks in the pattern is observed, which is proof of the successful formation of the aforementioned nanocatalyst. Scherrer’s formula was used to calculate the average particle size of the catalyst, *D* = 0.9λ/β cos θ, where λ is the wavelength of the incident X-ray (0.154 nm), β is the full width of the peak at half maxima, and θ is the Bragg’s angle.^48^ According to the spectrum related to the composite material mixed MOF-salinidol/Pd(II) 7, 2θ = 10–40° indicates the presence of a catalyst in the structure. Also, the XRD peaks in regions 42.64 (111), 50.09 (200), and 69.59 (220) degrees indicate the presence of Pd in the structure, which corresponds to cards 1043–46 from the JCPDS database. The calculated value of 90 nm agrees with the result obtained from SEM images.

### 3.2 Optimization Conditions of Alcohols Oxidation

In the first step, mixed MOF-Salinidol/Pd(II)7 was investigated to catalyze the aerobic oxidation of cyclohexanol as a model reaction (K_2_CO_3_ as base, 1 atm O_2_ filled flasks). The dehydrogenation and oxidation reactions of cyclohexanol to cyclohexanone under different weights of mixed MOF-salinidol/Pd(II) **7**, solvents, temperatures, and times were explored, and the obtained results are shown in Table 2. Various solvents such as H_2_O, toluene, and trifluorotoluene have been used, and triflourotoluene at 95 °C has been found to provide the most efficient dehydrogenation of cyclohexanol to cyclohexanone (entries 1–6). The model reaction was investigated by different loadings of mixed MOF-salinidol/Pd(II) **7**. When the reaction was investigated with 0.1 to 0.3 mol % catalysts, the product yields increased, and the reaction time decreased. Using more than 0.3 mol % of catalyst had no positive effect on the reaction efficiency (entries 7–10). Next, the base plays a vital influence in chemical conversions regarding reaction time and product yield. Reactions to determine the effect of a base on the model reaction showed that the reaction did not occur under base-free conditions, despite the extension of the reaction time (entry 11). Finally, to investigate the effect of functionalization, the model reaction was investigated with mixed MOF/Pd (II), and only a moderate yield was attained (entry 12). Investigation of reaction conditions in the absence of a catalyst clearly demonstrated the effect of a catalyst on the reaction efficiency (entry 13). The reaction was also studied with homogeneous Salinidol/Pd(II) catalysis, but only 6% conversion was obtained (entry 14).

**Table 2:**
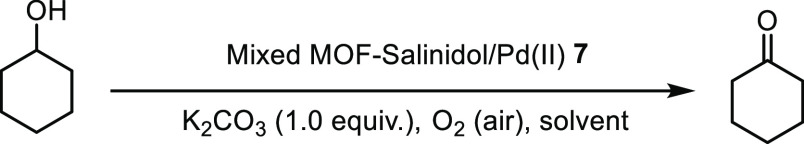
Optimization of the Reaction Conditions Using Mixed MOF-Salinidol/Pd(II) **7**

entry	solvent	temp (°C)	catalyst (mol %)	time (h)	yield [%]^a^
1	H_2_O	75	0.3	12	3
2	H_2_O	95	0.3	12	16
3	toluene	75	0.3	12	28
4	toluene	95	0.3	12	71
5	trifluorotoluene	75	0.3	12	73
**6**	**trifluorotoluene**	**95**	**0.3**	**1**	**>99**
7	trifluorotoluene	95	0.4	1	>99
8	trifluorotoluene	95	0.3	1	>99
9	trifluorotoluene	95	0.2	3	>99
10	trifluorotoluene	95	0.1	12	58
11	trifluorotoluene	95	0.3	1	38^b^
12	trifluorotoluene	95	0.3	6	60^c^
13	trifluorotoluene	95		12	0^d^
14	trifluorotoluene	95	0.3	12	6^e^

a Determined by GC.

b Without K_2_CO_3_.

c With mixed MOF/Pd(II).

d Without catalyst.

e Salinidol/Pd(II).

Under established conditions, the scope of the improved mixed MOF-salinidol/Pd (II) **7** catalyst was examined in the oxidation of a variety of alcohols, and the results are presented in Table 3. The results clearly show that the corresponding carbonyl compounds were obtained with great efficiency and selectivity. Aliphatic alcohols seem to require longer reaction times than aromatic ones (entries 4–7).

**Table 3:**
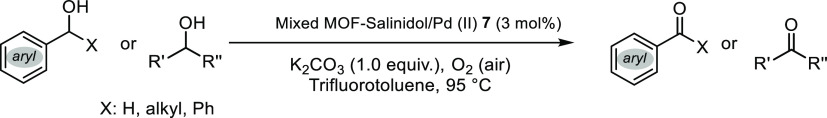

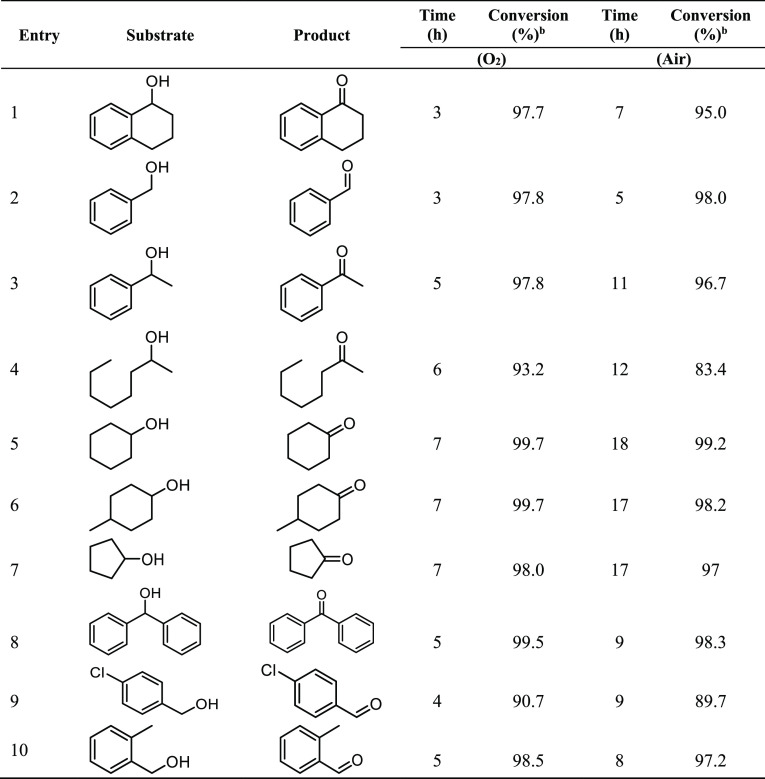
Verification of Various Variables for Reduction of Model Reaction Using Mixed MOF-Salinidol/Pd(II) Catalyst **7**^a^

a Reaction condition: alcohol (1.0 mmol), mixed MOF-salinidol/Pd(II) 7 (0.3 mol %), in trifluorotoluene (3 mL) at 95 °C for 1 h.

b Determined by GC.

The fact that aromatic structures are oxidized to the corresponding ketones in a shorter reaction time than aliphatic ones indicates that the π–π interaction between the aromatic ring and the catalyst support surface can bind to the adsorption and diffusion of alcohols more easily. Compared with aliphatic alcohols, these results can be assigned to the easier adsorption and diffusion of aromatic alcohols via π–π interaction between the aromatic ring of reactants and the catalyst support surface. This facilitates hydrogen bond formation between the −OH/–NH ends on the catalyst surface and the molecules. Prevention of the over-oxidation of benzyl alcohol to benzoic acid is still a serious challenge.^49^ But in all cases, full selectivity for aldehyde against carboxylic acid or other oxidizing products has been achieved. Benzyl alcohols are strongly affected by the electronic properties of the substituents. The benzyl alcohol was selectively converted to benzaldehyde in high yield after 3 h, while the 4-chlorobenzyl alcohol was oxidized to the corresponding aldehyde in good yield after 4 h. This reveals that the structures to which electron-withdrawing groups are attached slightly reduce the conversion rate and activity compared to benzyl alcohol. Accordingly, it can be said that electron-withdrawing groups have a negative effect on oxidation reaction conversion. The secondary alcohols were also altered to related carbonyl derivatives (entries 4–8). The reaction of the sterically demanding alcohol 1,2,3,4-tetrahydronaphthalen-1-ol proceeded to give the corresponding 3,4-dihydronaphthalen-1(2*H*)-one in both cases, and with molecular oxygen and air, it achieved an optimal efficiency of more than 95% (entry 1). The reaction also proceeds quite well under the atmosphere, indicating that there is no appreciable slowing down of the reaction by the concentration of dissolved oxygen in the solvent.

### 3.3 Optimization Conditions of Nitroarene Reduction

The final product of aniline resulting from the hydrogenation of nitrobenzene by simply using a hydrogen balloon with mixed MOF-salinidol/Pd(II) **7** was conducted in water under the reflux system equipped with a reflux condenser (Table 4). At first, the reaction was run for 12 h without a catalyst in the presence of water, and no conversion could be achieved (entry 1). When the reaction was performed in the presence of 0.05 mol % catalyst, a very good conversion was succeeded with 83% (entry 2). In the use of 0.1 mol % mixed MOF-salinidol/Pd(II) **7**, nitrobenzene was converted to aniline with 100% complete conversion (entry 3). Decreasing the temperature to 50 °C showed a slight decrease in product conversion (entry 4). The response of the studied model at different times showed that the highest yield and conversion were obtained at 1 h (entries 5–7). Finally, a maximum yield and conversion of 0.1 mol % Pd catalyst were achieved and after 1 h at 75 °C with H_2_ as reducing agent (entry 3). The reaction products were extracted and purified by diethyl ether after the catalytic cycle process was completed. The extraction of the reaction product from diethyl ether is very simple, and this solvent does not cause damage to the catalyst. Reaction progress was monitored by the TLC. The final product of aniline for verification of various variables was identified and measured by the GC technique.

**Table 4:**
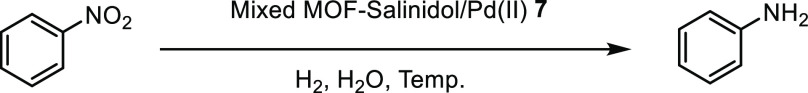
Optimization of the Nitroarene Reduction Reaction Conditions Using Mixed MOF-Salinidol/Pd(II) **7**

entry	*T* (°C)	Pd (mol %)	time (min)	conversion (%)^a^^,^^b^^,^^c^
1	75	0	60	N.R.
2	75	0.05	60	83
3	**75**	**0.10**	**60**	**100**
4	50	0.10	60	97
5	75	0.10	15	61
6	75	0.10	30	80
7	75	0.10	45	90

a Reaction conditions: nitrobenzene (1.0 mmol), mixed MOF-salinidol/Pd(II) (0.1 mol %), and solvent (10 mL).

b 1.0 mmol nitrobenzene was used.

c Determined by GC.

Many different substituted nitrobenzenes have been used to demonstrate the generality of the method. The expanded reaction scope and excellent reaction conversions were obtained with nitrobenzenes having electron-donating and electron-withdrawing groups. This reveals that the substituents in nitrobenzene have no noticeable effect on the reaction conversion (Table 5).^50^

**Table 5:**
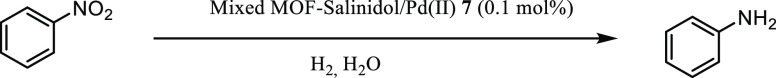

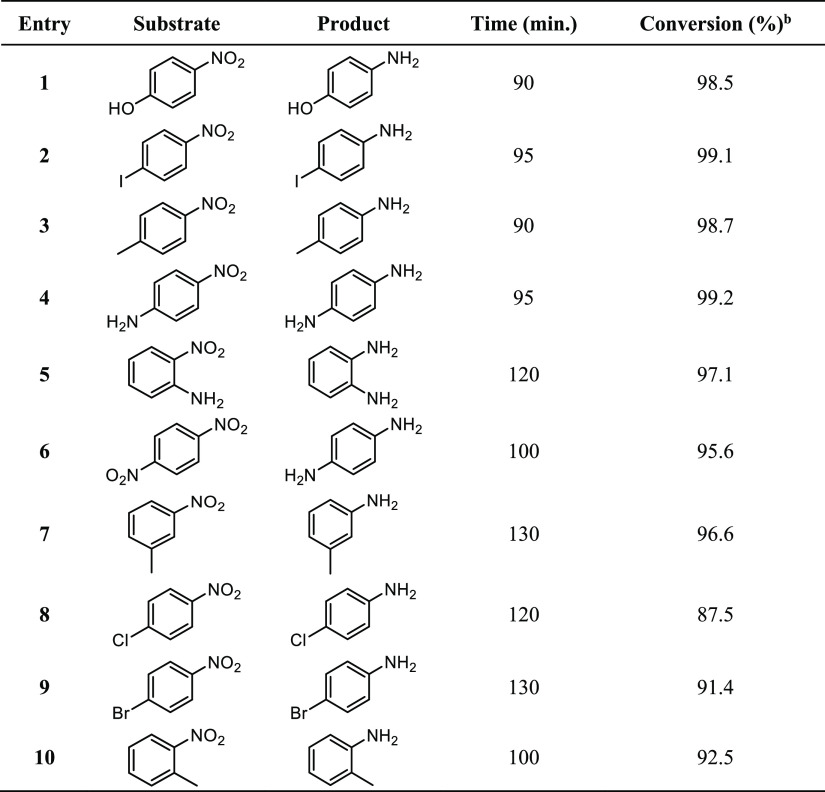
Hydrogenation Reaction for Nitrobenzene and Derivatives^a^

a H_2_O (10 mL), H_2_ (1 atm), nitroarenes (1.0 mmol), and mixed MOF-salinidol/Pd(II) **7** (1.0 mol %) at 75 °C.

b Determined by GC.

Finally, the catalytic efficiencies of the mixed MOF-salinidol/Pd(II) **7** were compared to the catalysts specified in Tables 6 and 7. The catalysts described for oxidation reactions suffer from several drawbacks, including the involvement of non-renewable catalysts, high temperatures, the use of reagents, precarious organic solvents, and the need for large quantities of catalysts. Compared to the reported alcohol oxidation catalysts, the mixed MOF-salinidol/Pd(II) **7** catalyst showed more acceptable performance. The high porosity surface area and immobilization of the Pd complex in the open framework of MOFs lead to this good performance. So, our work demonstrates the potential that MOFs present to metallic anchor complexes to transform homogeneous catalysis into a heterogeneous process (entry 7).

**Table 6:**
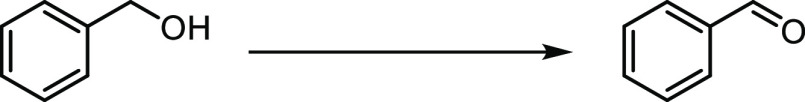
Comparison of the Catalytic Performance of the Mixed MOF-Salinidol/Pd(II) **7** with Some Other Catalysts in the Oxidation Reaction of Alcohols

entry	reaction condition	*T* (°C)	time (h)	yield (%)	refs
1	CuPc nanoparticle 5 mol %, *n*-Bu_4_NHSO_5_ (0.7 g), H_2_O	85	168	79	(51)
2	Fe(II) phthalocyanines/TBHP(500/1)	70	3	72	(52)
3	Al-MCM-41(10)-CuPc (0.055 g)	100	4	48	(53)
4	CoPc@Cell (0.05 g), KOH, *o*-xylene, O_2_	r.t.	8.5	83	(54)
5	MIL-101-NH_2_/CoTSPc (0.42 mol %), *p*-xylene, KOH, air (300 mL min^–1^)	100	8	95	(55)
6	TACoPc/Si–Cl (20 mg), H_2_O_2_, CH_3_CN, visible light	r.t.	6	60	(56)
7	mixed MOF-salinidol/Pd(II) (0.3 mol %)	95	1	>99	this work

**Table 7:**
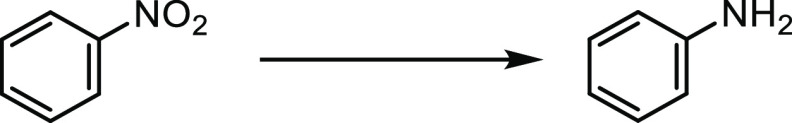
Comparing the Catalytic Performance of the Mixed MOF-Salinidol/Pd(II) **7** with Other Catalysts in the Hydrogenation Reaction of Nitrobenzene

entry	reaction condition	*T* (°C)	time (min)	yield (%)	refs
1	Co@NMC-800, EtOH, H_2_	80	120	97.8	(57)
2	Pd/PEG catalyst, H_2_	r.t.	180	100	(58)
3	Pd/C/CNT, methanol	50	1500	100	(59)
4	Pd/Fe_2_O_3_, H_2_	r.t.	120	100	(60)
5	Pd@CQD@Fe_3_O_4_, EtOH/H_2_O, NaBH_4_	r.t.	240	90	(61)
6	Pd/C (5 wt %), EtOH, HCO_2_NH_4_	r.t.	720	99	(62)
7	RGO-Pd (Co), EtOH/H_2_O, NaBH_4_	r.t.	5	98	(63)
8	Cu/Co/Fe_2_O_4_–G, EtOH/H_2_O, NaBH_4_	50	13	99	(64)
9	Pd/COF-BASU2, EtOH/H_2_O, H_2_	75	60	100	(65)
10	mixed MOF-salinidol/Pd(II) **7**, EtOH/H_2_O, H_2_	75	60	100	this work

Furthermore, the catalytic efficiencies of the mixed MOF-Salinidol/Pd(II) **7** were compared with other reported catalysts for the reduction of nitrobenzene (Table 7). The reduction of nitrobenzene in the presence of mixed MOF-salinidol/Pd(II) **7** was carried out under conditions that can be considered an environmentally friendly method in water at an acceptable temperature and with a short reaction time. The synthesized nanocatalysts are very flexible at different temperatures and times. They are also very stable and conveniently insulating (entry 10).

The present catalyst system exhibits high catalytic activity even for the aerobic oxidation of alcohols under air rather than pure oxygen, affording the corresponding carbonyl products in high yields and with remarkable selectivities.

**Figure 7:**
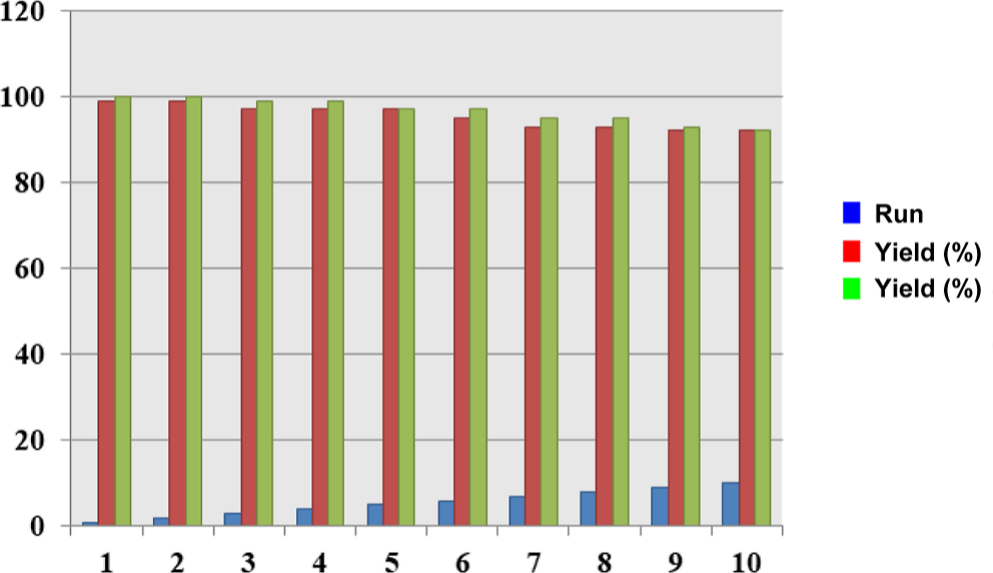
Recycling experiment of the mixed MOF-salinidol/Pd(II) **7** for benzyl alcohol oxidation (green) and hydrogenation reaction for nitrobenzene (red).

The catalyst used for the reaction was filtered through the reaction mixture and heated at 120 °C for 4 h. The recovered catalyst was reused ten times and provided excellent and comparable activity under the same reaction conditions (Figure 7).

**Figure 8:**
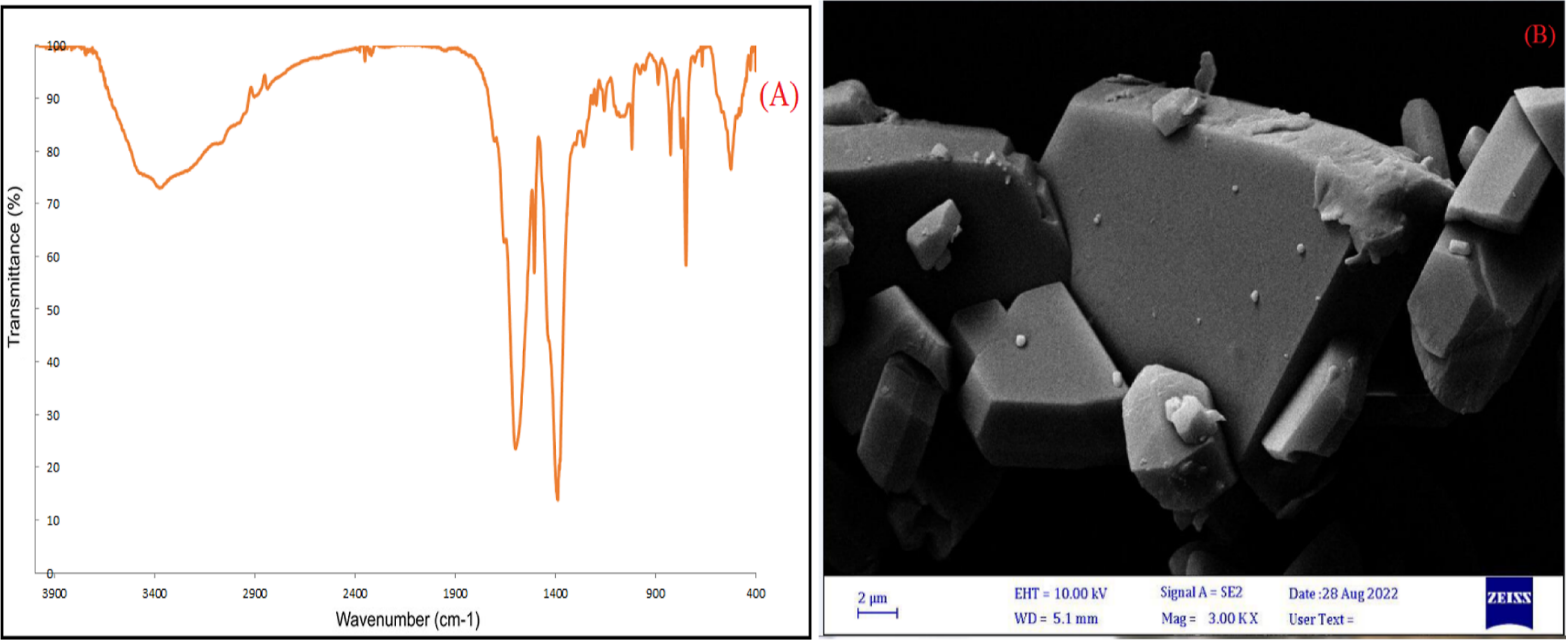
(A) FT-IR spectrum and (B) SEM images of mixed MOF-salinidol/Pd(II) **7** after using these catalysts ten times.

The recovery of the mixed MOF-salinidol/Pd(II) **7** catalyst demonstrates a highly efficient sequential application. An investigation of the FT-IR spectrum (Figure 8A) and SEM image (Figure 8B) after recovery shows the chemical/thermal stability of the synthesized catalyst. The Pd load in the material was found to be 10.60% and, after recovery, 10.57%, being estimated *via* the ICP-OES method.

## 4 Conclusions

This article reports an efficient and clean oxidation/reduction conversion protocol using mixed MOF-salinidol/Pd(II) **7** as a novel heterogeneous catalyst. Various analytical techniques, including powder X-ray diffraction, field emission scanning electron microscopy, EDX, BET, TGA, and FT-IR analyses, characterized the new materials based on MOF nano-composites. These materials showed considerably higher catalytic activity compared to typical Pd heterogeneous catalysts. Moreover, simple handling and work-up, low cost, mild reaction conditions, higher yields, high thermal stability, and exceptional catalyst reusability provide new opportunities to design new catalysts with improved performance for industrial and practical applications.

## Supporting Information Available

Mechanism of alcohol oxidation; proposed reaction pathway; GC spectra for oxidation of alcohols; and chromatogram output (PDF)
