# Supplementary material for: Pd-Coordinated Salinidol-Modified Mixed MOF: An Excellent Active Center for Efficient Nitroarenes Reduction and Selective Oxidation of Alcohols
Source: ACS Omega. 2023 Jun 7;8(24):22138–49. doi: 10.1021/acsomega.3c02414 (PMC10285956; doi:10.1021/acsomega.3c02414)
Supplement: Supplementary file 1 — ao3c02414_si_001.pdf [file ao3c02414_si_001.pdf]

Supporting Information

Pd Coordinated Salinidol-modified Mixed MOF: An Excellent Active Centre for Efficient Nitroarenes Reduction and Selective Oxidation of Alcohols

Hassan Keypour[a], Jamal Kouhdareh[a], Sedigheh Alavinia[b], Rahman Karimi-Nami[c]*, İdris Karakaya[d]**

[a] Department of Inorganic Chemistry, Faculty of Chemistry, Bu-Ali Sina University Hamedan, 6517838683, Iran

[b] Department of Organic Chemistry, Faculty of Chemistry, Bu-Ali Sina University Hamedan

[c] Department of Chemistry, Faculty of Science, University of Maragheh, Maragheh, Iran

[d] Department of Chemistry, College of Basic Sciences, Gebze Technical University, 41400 Gebze, Turkey

E-mail: karakaya@gtu.edu.tr

1. Materials and methods

IR spectra were taken by a FT-IR Shimadzu of the Nicolet Instrument Corporation spectrophotometer. XRD powder patterns were acquired on a XRD Rigaku Ultima IV X-ray diffractometer with a copper anode and a graphite monochromator using Cu Kα radiation, taking data from $2\theta = 2.5°$ to 80° at a scan rate of 0.03°/s and operating parameters of 40 kV and 80 mA. Thermal behavior was determined by Thermo Gravimetric Analysis (TGA) which was carried out using a Mettler Toledo TGA/STDA 851e. Samples (10 mg) placed in 70 µL alumina pans were heated under airflow of 40 mL/min between 35 and 600 °C with a heating rate of 10 °C/min. Scanning electron microscopy (SEM) images and energy-dispersive X-ray spectroscopy (EDX) were taken on a FEI NOVA Nano SEM 450 thermal field emission scanning electron microscope. Brunauer-Emmett-Teller (BET) measurements were obtained on a Beckman Coulter SA3100 plane area analysis. Graphs related to Gas Chromatography with the help of the device Gas Chromatography Agilent 6890 GC Series controlled by Agilent OpenLab software is an integrated suite to capture, analyze. Thin layer chromatography on precoated silica gel fluorescent 254 nm (0.2 mm) were used for monitoring the reaction progresses. The column used GC Capillary Column Rtx-1301 30m, 0.53mm ID, 3µ (G43). The volume of injection is equal to 1 µL and the concentration of samples is equal to 1 mg/ml in water.

2. Hot filtration test

To determine if Mixed MOF-Salinidol/Pd(II) **7** was functioning heterogeneousl, or if it was simply a reservoir for the more active soluble form of Pd, several heterogeneity tests were performed. First, the reaction of cyclohexanol was carried out in the presence of the catalyst Mixed MOF-Salinidol/Pd(II) **7**, 1 h until 66% conversion was reached. The solid was then filtered hot and transferred to another Schlenk flask containing K_2CO_3 at 95 °C under an O_2 atmosphere. The catalyst-free solution was then allowed to continue reacting, but no further reaction occurred after 24 h.

3. Plausible reaction mechanism of alcohol oxidation

The possible mechanism for selective alcohol oxidation using a palladium-based catalyst is presented in Scheme S1. The reaction may go forward at first by the oxidative addition of alcohol that leads to the synthesis of intermediate **A**. Next, intermediate **A** converted to intermediate **B** via deprotonation. Eventually, ß-hydride elimination of intermediate **B** leads to the desired product and regenerates the catalyst.

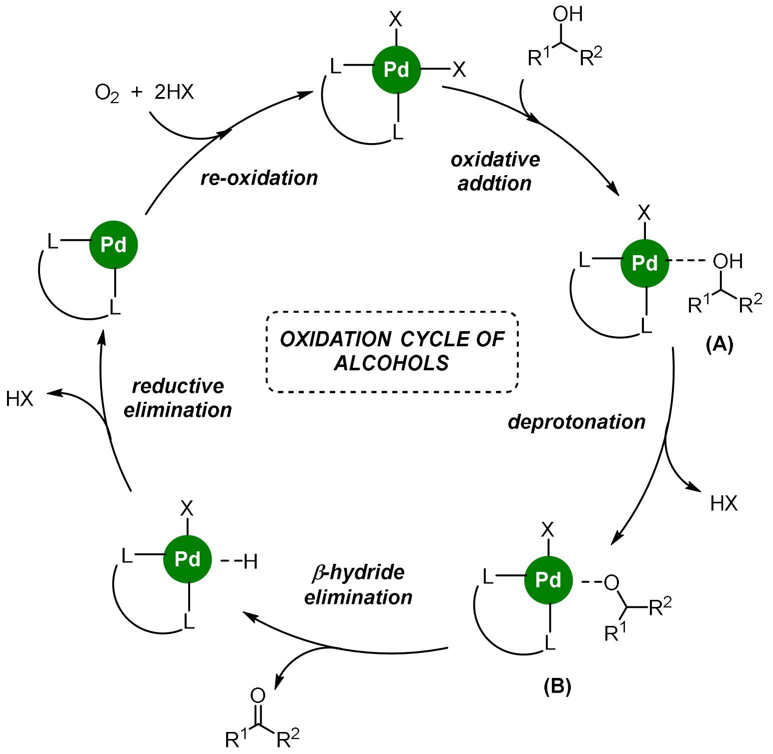

Scheme S1. Mechanism of alcohol oxidation using Mixed MOF-Salinidol/Pd(II) **7**

Scheme S2 presents the general mechanism of controlled hydrogenation of nitrobenzene in the presence of Mixed MOF-Salinidol/Pd(II) **7**. In consonance with this mechanism, the active

surface of palladium appears to bind hydrogen molecules and initiate a catalytic cycle with nitroarene (Scheme 2).

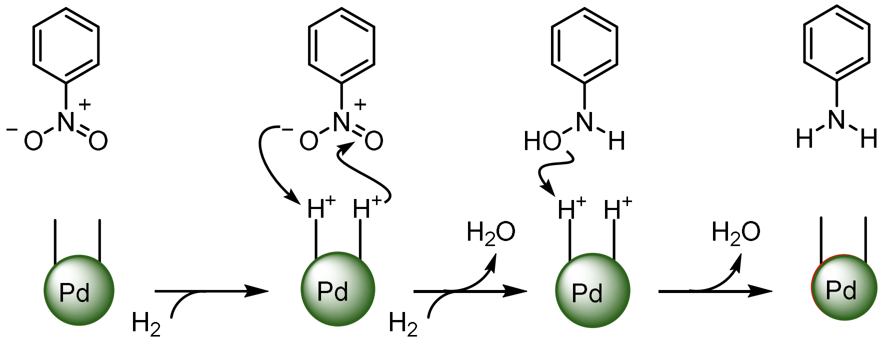

Scheme S2. Proposed reaction pathway for hydrogenation of nitrobenzene using Mixed MOF-Salinidol/Pd(II) **7**

3. The GC Spectra for Oxidation of Alcohols

```
Additional Info : Peak(s) manually integrated
```

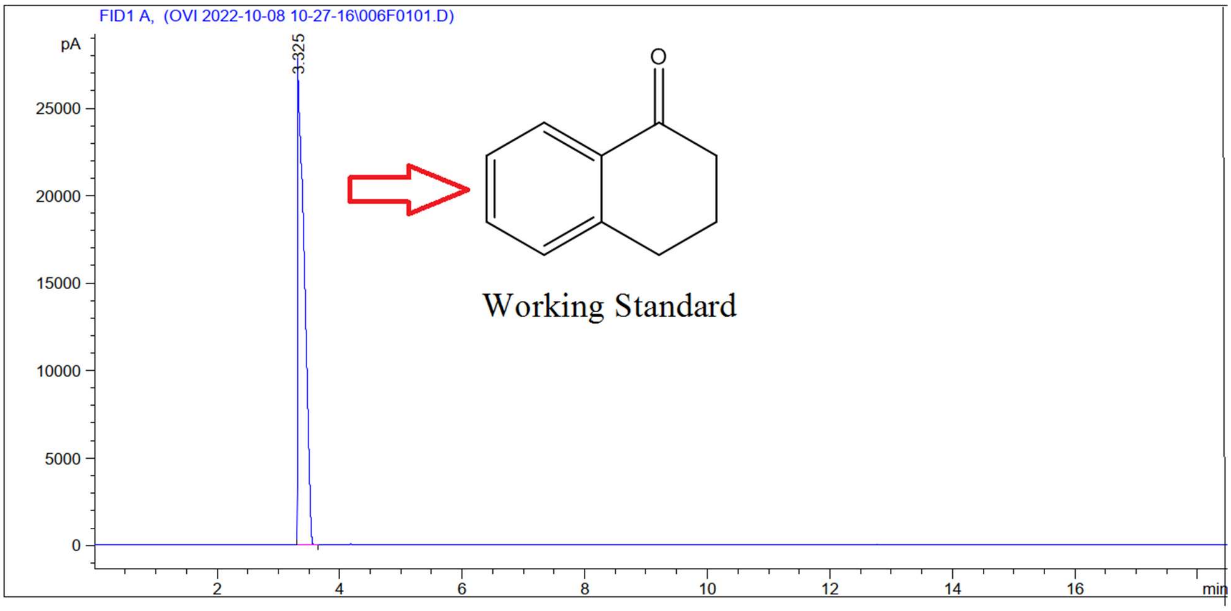


```
Peak RetTime  Type   Width     Area      Area    Name
  #   [min]          [min]    [pA*s]       %
----|-------|------|-------|----------|--------|---------------------
   1   3.325 BB S    0.0905 2.04368e5  1.000e2

Totals :                    2.04368e5
```

Catalyst	Substrate	Product	Catalyst ratio (mol%)	Retention time (RT) (min)	Area	Purity (%)
**N.A**	N.A	3,4-dihydronaphthalen-1(2H)-one	N.A	3.225	204368	100

Figure S1. Chromatogram output for 3,4-dihydronaphthalen-1(2H)-one working standard

Additional Info : Peak(s) manually integrated

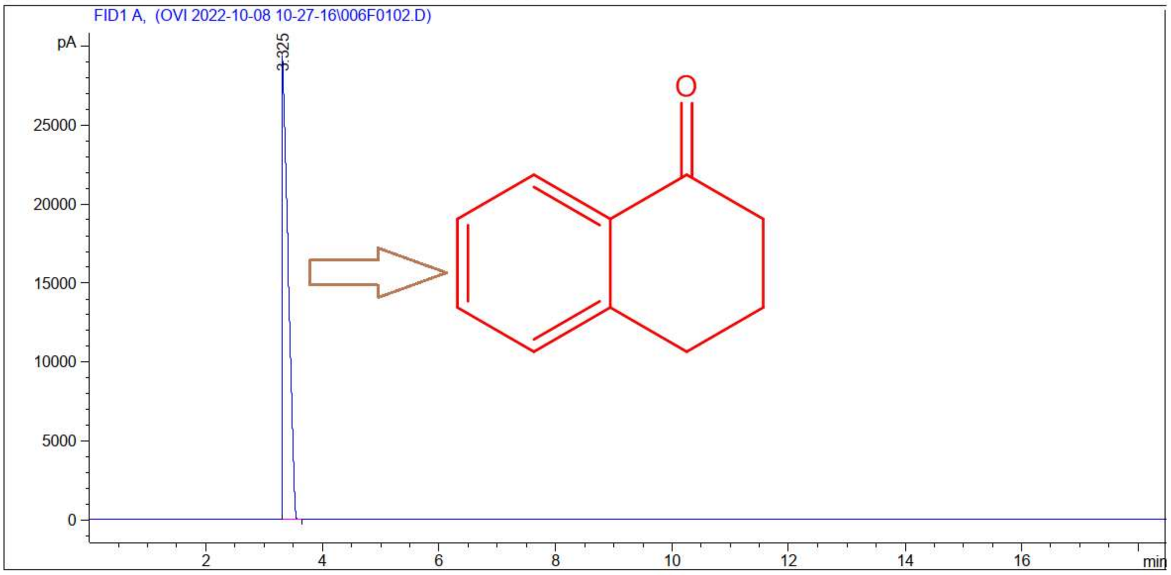


```
Peak RetTime  Type   Width    Area      Area    Name
  #   [min]          [min]   [pA*s]      %
----|-------|------|-------|----------|--------|---------------------
   1   3.325 BB S    0.0851 1.99757e5  1.000e2

Totals :                    1.99757e5
```

Catalyst	Substrate	Product	Catalyst ratio (mol%)	Retention time (RT) (min)	Area	Substrate conversion (%)
**Mixed MOF-Salinidol/Pd(II)**	1,2,3,4-tetrahydronaphthalen-1-ol	3,4-dihydronaphthalen-1(2H)-one	0.3	3.225	199757	97.74

Figure S2. Chromatogram output for 3,4-dihydronaphthalen-1(2H)-one result of oxidation with Molecular Oxygen

```
Additional Info : Peak(s) manually integrated
```

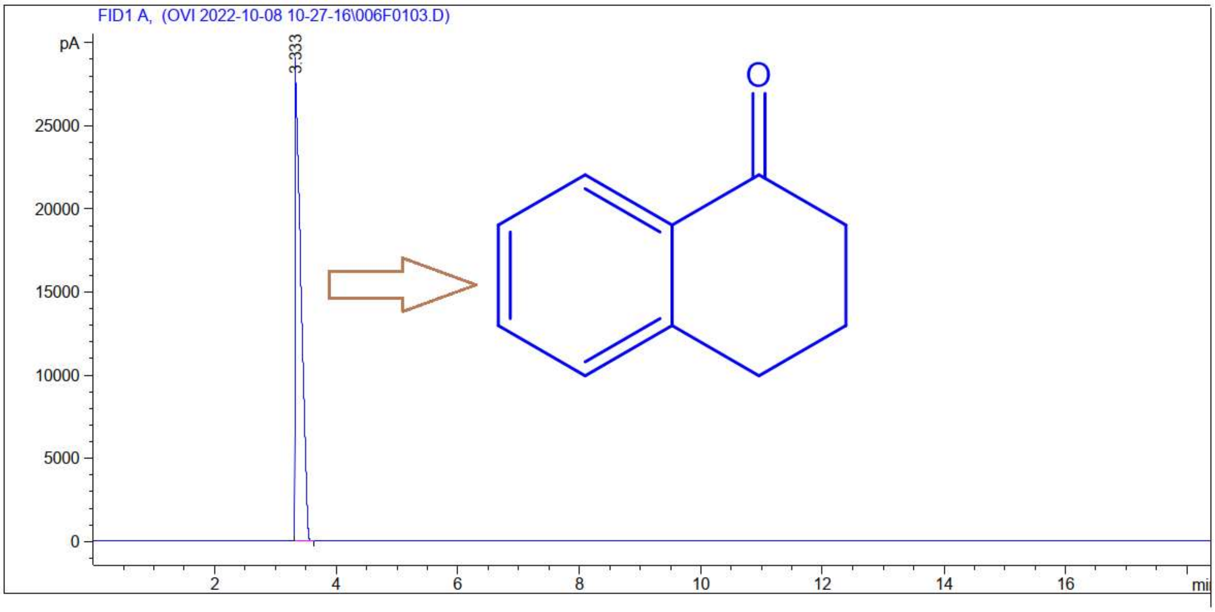


```
Peak RetTime  Type   Width     Area      Area     Name
  #   [min]          [min]   [pA*s]       %
----|-------|------|-------|----------|--------|---------------------
   1   3.333 BB S    0.0874 1.94134e5  1.000e2

Totals :                    1.94134e5
```

Catalyst	Substrate	Product	Catalyst ratio (mol%)	Retention time (RT) (min)	Area	Substrate conversion (%)
**Mixed MOF-Salinidol/Pd(II)**	1,2,3,4-tetrahydronaphthalen-1-ol	3,4-dihydronaphthalen-1(2H)-one	0.3	3.233	194134	94.99

Figure S3. Chromatogram output for 3,4-dihydronaphthalen-1(2H)-one result of oxidation with Air

Additional Info : Peak(s) manually integrated

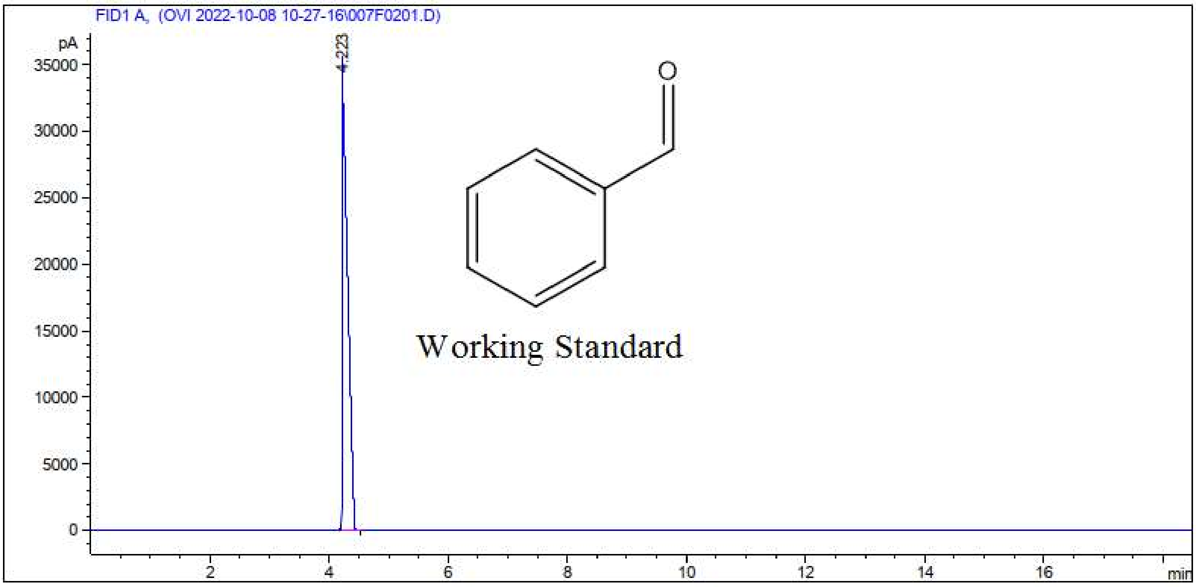


```
Signal 1: FID1 A,

Peak RetTime  Type   Width     Area      Area    Name
  #   [min]          [min]    [pA*s]       %
----|-------|------|-------|----------|--------|---------------------
   1   3.325          0.0000    0.00000  0.00000
   2   4.223 VB S     0.1115 2.36897e5  1.000e2  ?

Totals :                     2.36897e5
```

Catalyst	Substrate	Product	Catalyst ratio (mol%)	Retention time (RT) (min)	Area	Purity (%)
**NA**	NA	benzaldehyde	NA	4.223	236897	100

Figure S4. Chromatogram output for benzaldehyde working standard

Additional Info : Peak(s) manually integrated

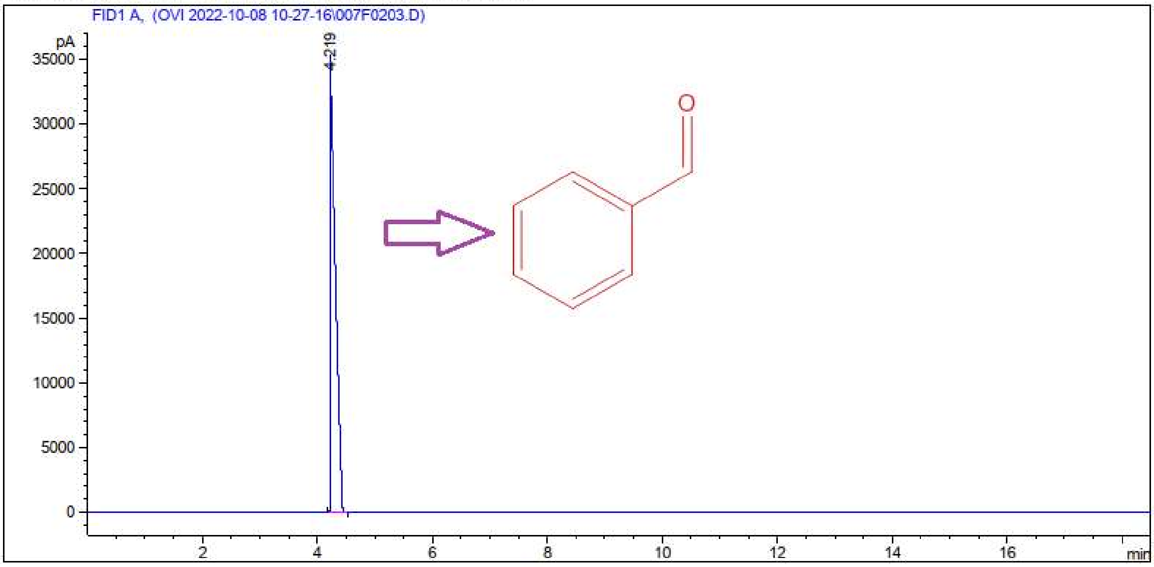


```
Peak RetTime  Type   Width     Area      Area    Name
  #   [min]          [min]    [pA*s]       %
----|-------|------|-------|----------|--------|---------------------
   1   3.325         0.0000    0.00000  0.00000
   2   4.219 BB S    0.0852 2.31822e5  1.000e2  ?

Totals :                    2.31822e5
```

Catalyst	Substrate	Product	Catalyst ratio (mol%)	Retention time (RT) (min)	Area	Substrate conversion (%)
**Mixed MOF-Salinidol/Pd(II)**	phenylmethanol	benzaldehyde	0.3	4.219	231822	97.85

Figure S5. Chromatogram output for benzaldehyde result of oxidation with Molecular Oxygen

```
Additional Info : Peak(s) manually integrated
```

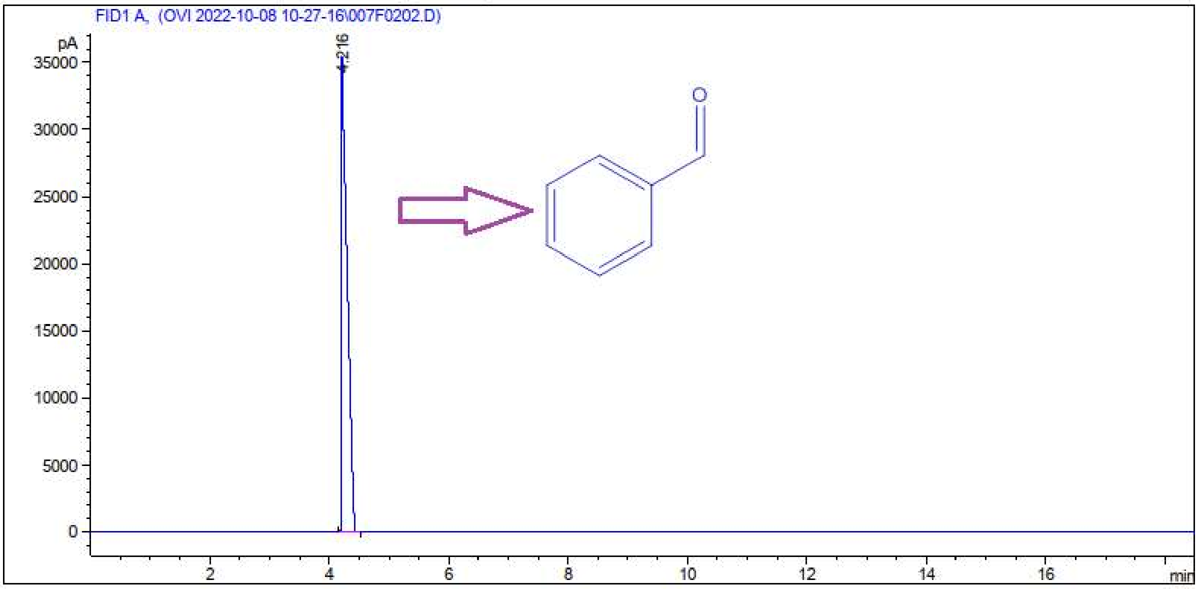


```
Signal 1: FID1 A,

Peak RetTime  Type   Width     Area      Area    Name
  #   [min]          [min]    [pA*s]      %
----|-------|------|-------|----------|--------|---------------------
   1   3.325          0.0000    0.00000   0.00000
   2   4.216 BB S     0.0812 2.28999e5  1.000e2   ?

Totals :                    2.28999e5
```

Catalyst	Substrat e	Product	Catalyst ratio (mol%)	Retention time (RT) (min)	Area	Substrate conversion (%)
**Mixed MOF-Salinidol/Pd(II)**	phenylm ethanol	benzaldehyd e	0.3	4.216	228999	96.66

Figure S6. Chromatogram output for benzaldehyde result of oxidation with Air

```
Additional Info : Peak(s) manually integrated
```

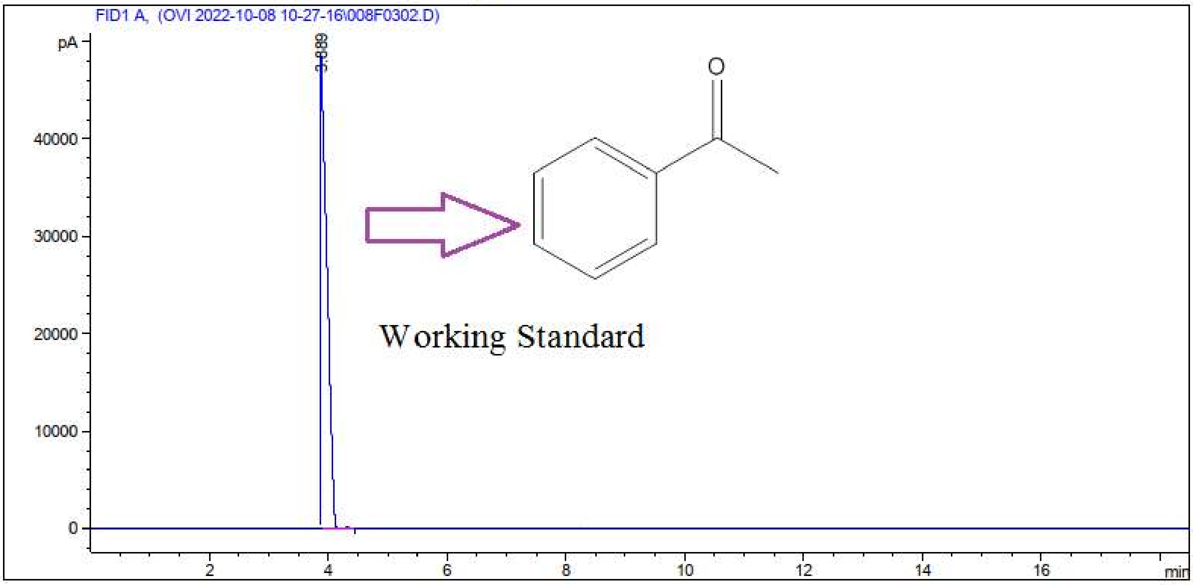


```
Signal 1: FID1 A,

Peak RetTime  Type   Width     Area      Area    Name
  #   [min]          [min]    [pA*s]       %
----|-------|------|-------|----------|--------|---------------------
   1   3.889 VB S    0.1184 3.39737e5  1.000e2  ?

Totals :                    3.39737e5
```

Catalyst	Substrate	Product	Catalyst ratio (mol%)	Retention time (RT) (min)	Area	Purity (%)
**NA**	NA	acetophenone	NA	3.899	339737	100

Figure S7. Chromatogram output for acetophenone working standard

Additional Info : Peak(s) manually integrated

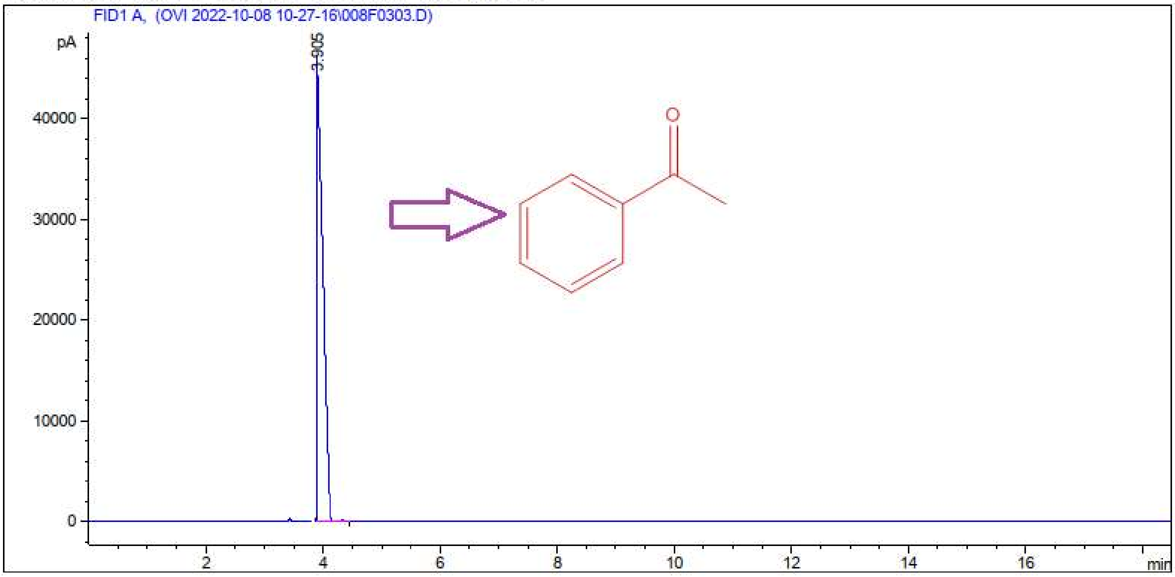


```
Signal 1: FID1 A,

Peak RetTime  Type   Width     Area      Area    Name
  #   [min]          [min]    [pA*s]      %
----|-------|------|-------|----------|--------|---------------------
   1   3.905 VB S    0.1164 3.16650e5  1.000e2  ?

Totals :                    3.16650e5
```

Catalyst	Substrate	Product	Catalyst ratio (mol%)	Retention time (RT) (min)	Area	Substrate conversion (%)
**Mixed MOF-Salinidol/Pd(II)**	1-phenylethanol	acetophenone	0.3	3.905	316650	93.2

Figure S8. Chromatogram output for acetophenone result of oxidation with Molecular Oxygen

Additional Info : Peak(s) manually integrated

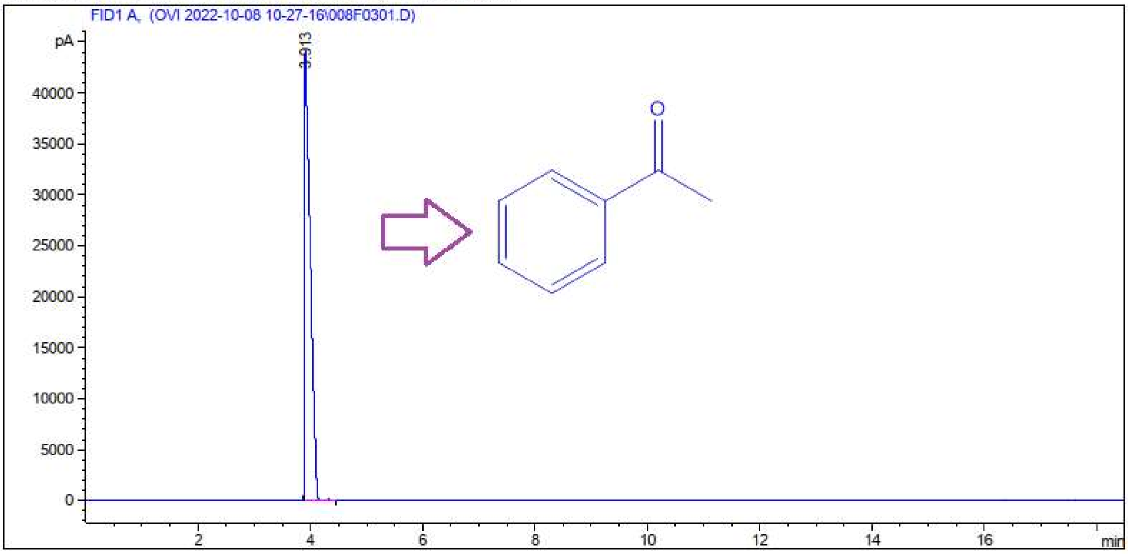


```
Signal 1: FID1 A,

Peak RetTime  Type   Width     Area       Area    Name
  #    [min]          [min]    [pA*s]       %
----|--------|------|-------|----------|--------|---------------------
   1   3.325          0.0000    0.00000  0.00000
   2   3.913 VB S     0.1061 2.83345e5  1.000e2   ?

Totals :                     2.83345e5
```

Catalyst	Substrate	Product	Catalyst ratio (mol%)	Retention time (RT) (min)	Area	Substrate conversion (%)
**Mixed MOF-Salinidol/Pd(II)**	1-phenylethanol	acetophenone	0.3	3.913	283345	83.4

Figure S9. Chromatogram output for acetophenone result of oxidation with Air

Additional Info : Peak(s) manually integrated

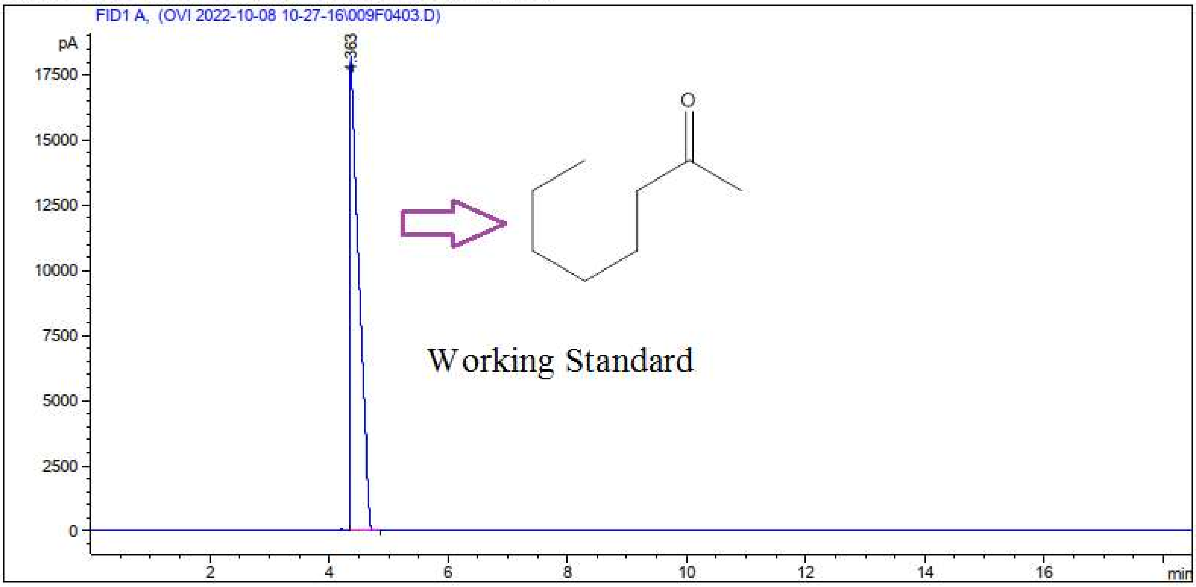


```
Peak RetTime  Type   Width      Area      Area    Name
  #   [min]          [min]    [pA*s]       %
----|-------|------|-------|----------|--------|---------------------
   1   4.363 BB S    0.1169 1.77281e5  1.000e2  ?


Totals :                    1.77281e5
```

Catalyst	Substrate	Product	Catalyst ratio (mol%)	Retention time (RT) (min)	Area	Purity (%)
**NA**	NA	octan-2-one	NA	4.363	177281	100

Figure S10. Chromatogram output for octan-2-one working standard

```
Additional Info : Peak(s) manually integrated
```

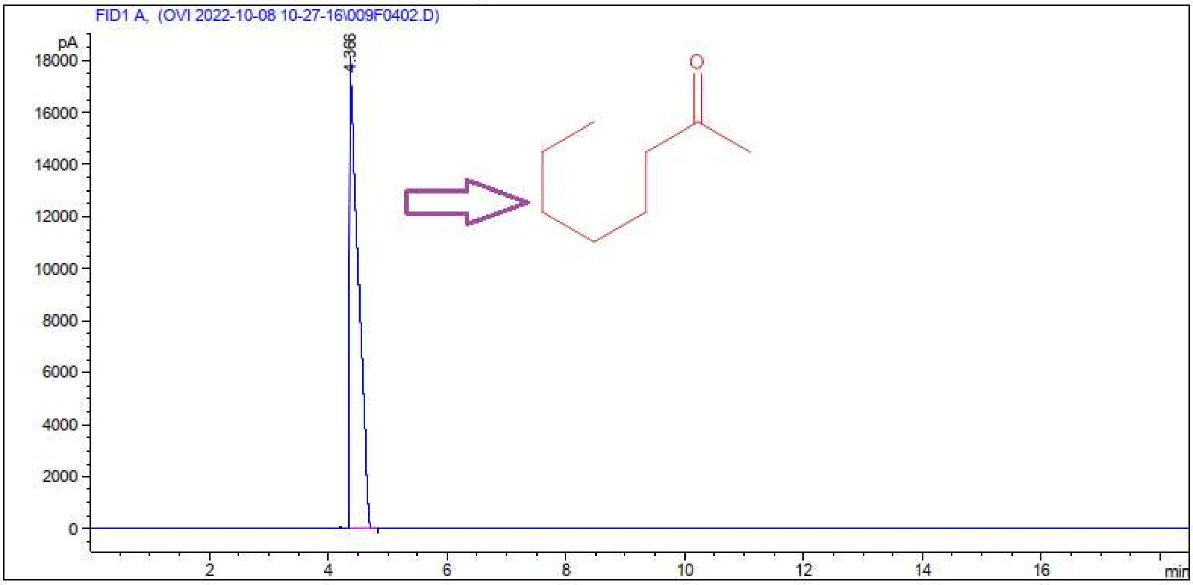


```
Signal 1: FID1 A,

Peak RetTime  Type   Width     Area      Area    Name
  #   [min]          [min]    [pA*s]       %
----|-------|------|-------|----------|--------|---------------------
   1   4.366 BB S    0.1218 1.76767e5  1.000e2  ?


Totals :                    1.76767e5
```

**Catalyst**	**Substrate**	**Product**	**Catalyst ratio (mol%)**	**Retention time (RT) (min)**	**Area**	**Substrate conversion (%)**
**Mixed MOF-Salinidol/Pd(II)**	octan-2-ol	octan-2-one	0.3	4.366	176767	99.71

Figure S11. Chromatogram output for octan-2-one result of oxidation with Molecular Oxygen

Additional Info : Peak(s) manually integrated

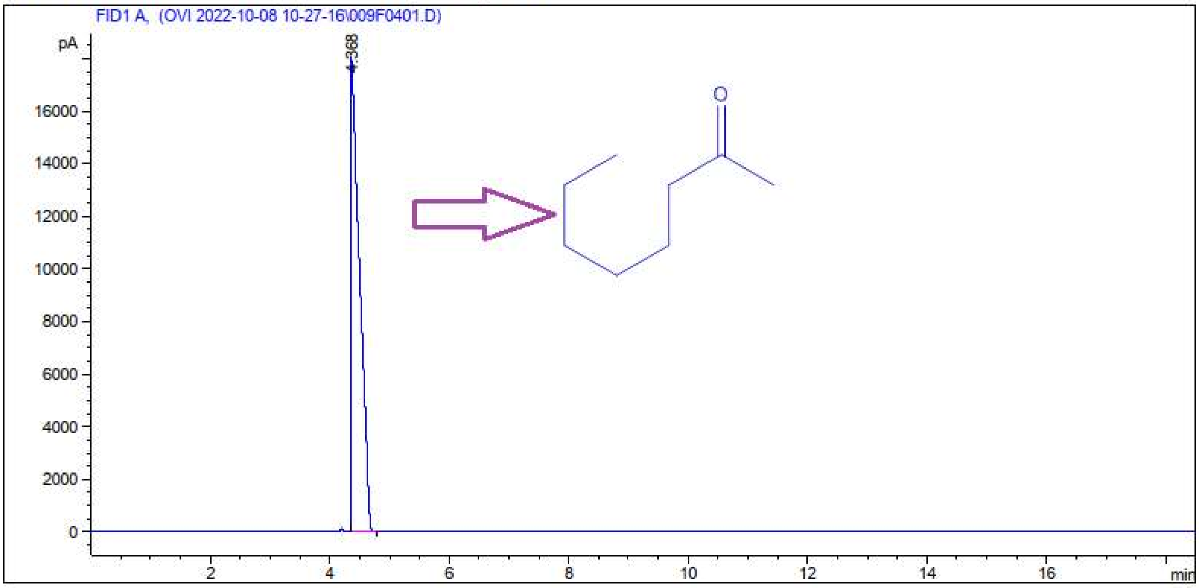


```
Signal 1: FID1 A,

Peak RetTime  Type   Width     Area      Area    Name
  #   [min]          [min]    [pA*s]       %
----|-------|------|-------|----------|--------|---------------------
   1   4.368 BB S    0.1192 1.75934e5  1.000e2  ?

Totals :                    1.75934e5
```

**Catalyst**	**Substrate**	**Product**	**Catalyst ratio (mol%)**	**Retention time (RT) (min)**	**Area**	**Substrate conversion (%)**
**Mixed MOF-Salinidol/Pd(II)**	octan-2-ol	octan-2-one	0.3	4.368	175934	99.24

Figure S12. Chromatogram output for octan-2-one result of oxidation with Air

```
Additional Info : Peak(s) manually integrated
```

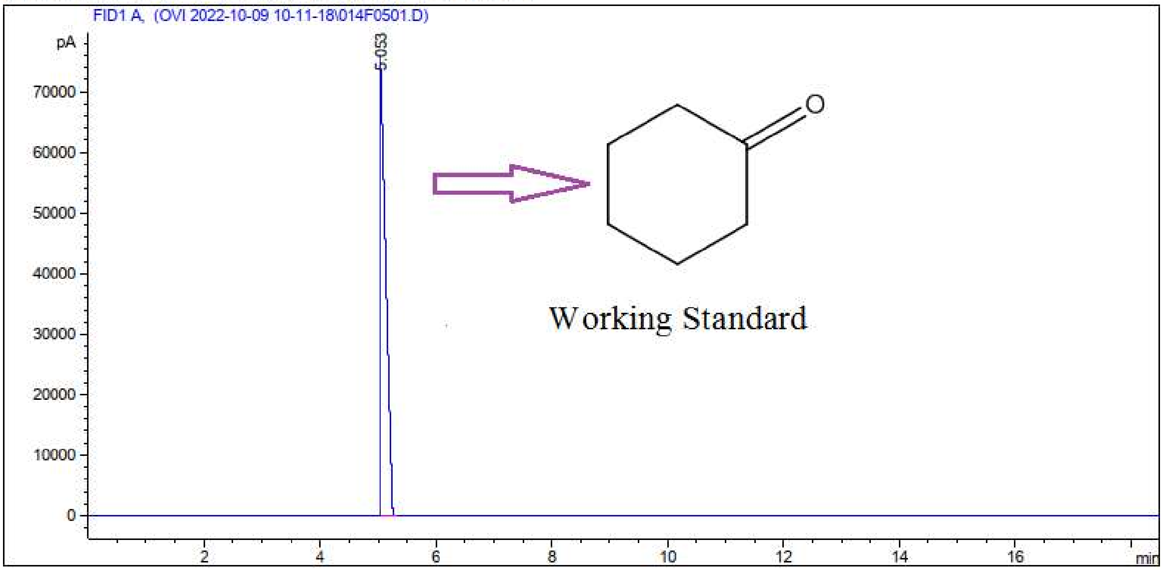


```
Signal 1: FID1 A,

Peak RetTime  Type   Width     Area      Area    Name
  #   [min]          [min]    [pA*s]      %
----|-------|------|-------|----------|--------|---------------------
   1   5.863 VB S    0.0505 1.45740e4   2.81248 ?
```

Catalyst	Substrate	Product	Catalyst ratio (mol%)	Retention time (RT) (min)	Area	Purity (%)
**NA**	NA	cyclohexanone	NA	5.863	145740	100

Figure S13. Chromatogram output for cyclohexanone working standard

Additional Info : Peak(s) manually integrated

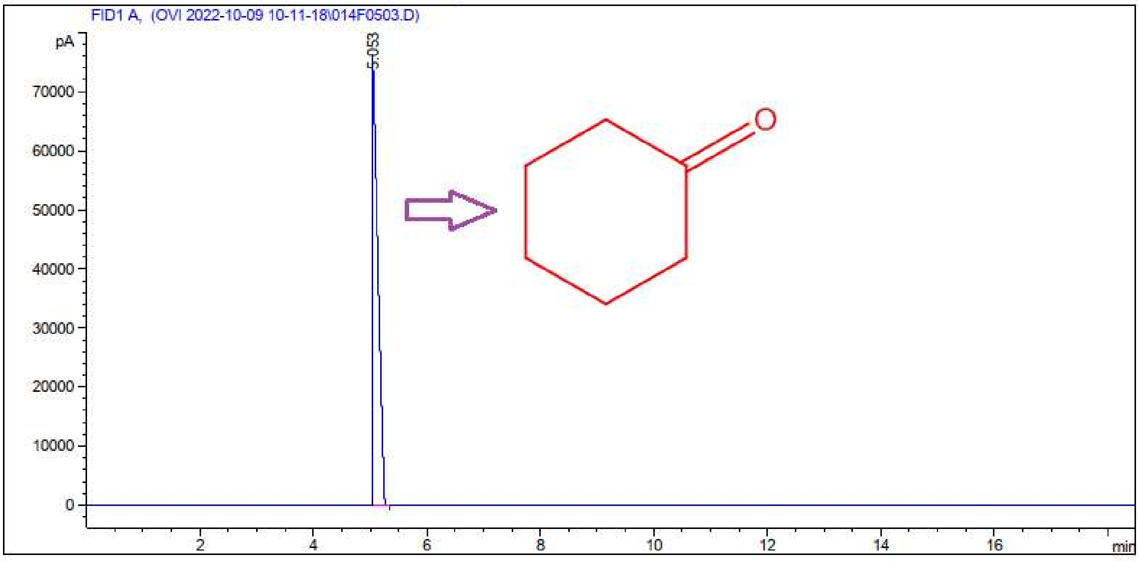


```
Signal 1: FID1 A,

Peak RetTime  Type   Width     Area      Area    Name
  #   [min]          [min]   [pA*s]        %
----|-------|------|-------|----------|--------|---------------------
   1   5.863 VB S    0.0501 1.45318e4   2.80834 ?
```

Catalyst	Substrate	Product	Catalyst ratio (mol%)	Retention time (RT) (min)	Area	Substrate conversion (%)
**Mixed MOF-Salinidol/Pd(II)**	cyclohexanol	cyclohexanone	0.3	5.863	145318	99.71

Figure S14. Chromatogram output for cyclohexanone result of oxidation with Molecular Oxygen

```
Additional Info : Peak(s) manually integrated
```

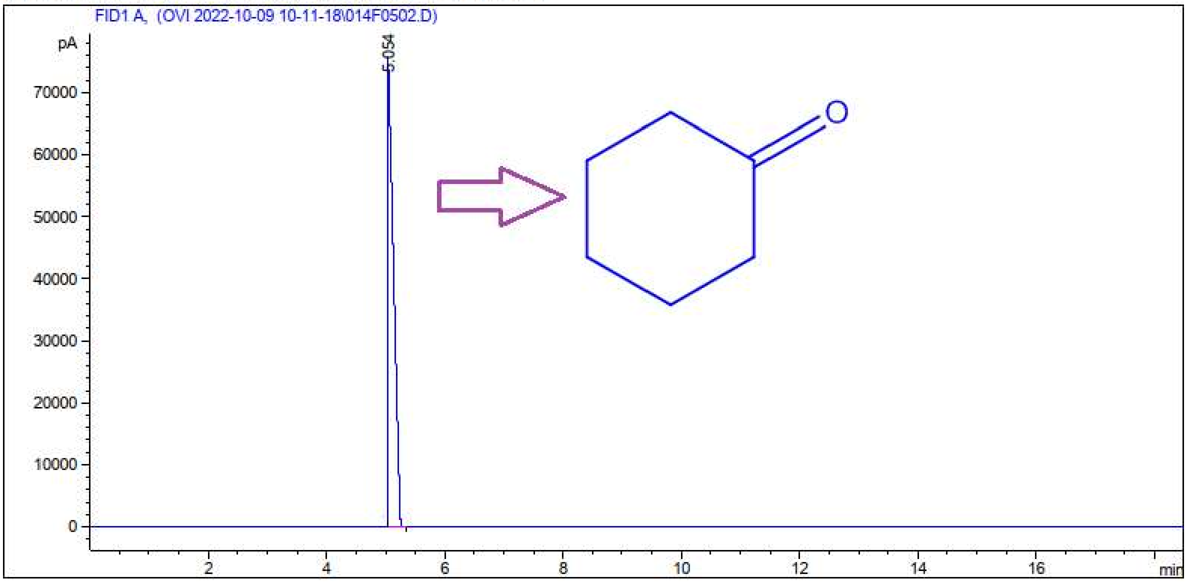


```
Signal 1: FID1 A,

Peak RetTime  Type   Width     Area      Area    Name
  #   [min]          [min]   [pA*s]       %
----|-------|------|-------|----------|--------|---------------------
   1   5.863 VB      0.0504 1.43123e4   2.80927 ?
```

Catalyst	Substrate	Product	Catalyst ratio (mol%)	Retention time (RT) (min)	Area	Substrate conversion (%)
**Mixed MOF-Salinidol/Pd(II)**	cyclohexanol	cyclohexanone	0.3	5.863	143123	98.2

Figure S15. Chromatogram output for cyclohexanone result of oxidation with Air

Additional Info : Peak(s) manually integrated

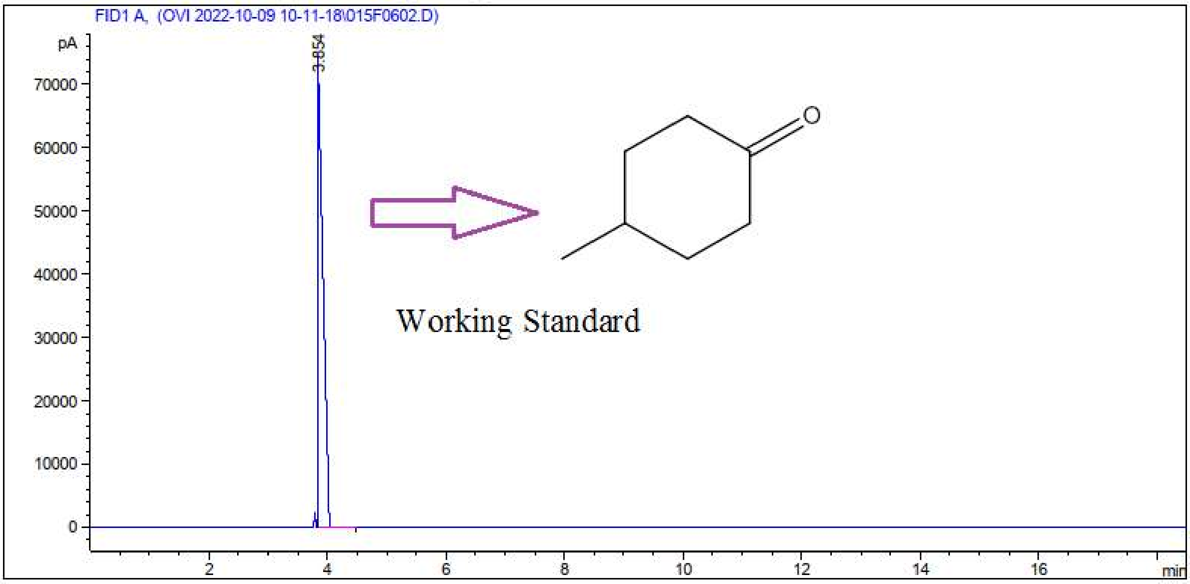


```
Signal 1: FID1 A,

Peak RetTime  Type   Width     Area      Area     Name
  #    [min]          [min]    [pA*s]      %
----|-------|------|-------|----------|--------|---------------------
   1   3.854 VB S    0.0984 4.29917e5  80.31337 ?
```

Catalyst	Substrate	Product	Catalyst ratio (mol%)	Retention time (RT) (min)	Area	Purity (%)
**NA**	NA	4-methylcyclohexanone	NA	3.854	429917	100

Figure S16. Chromatogram output for 4-methylcyclohexanone working standard

```
Additional Info : Peak(s) manually integrated
```

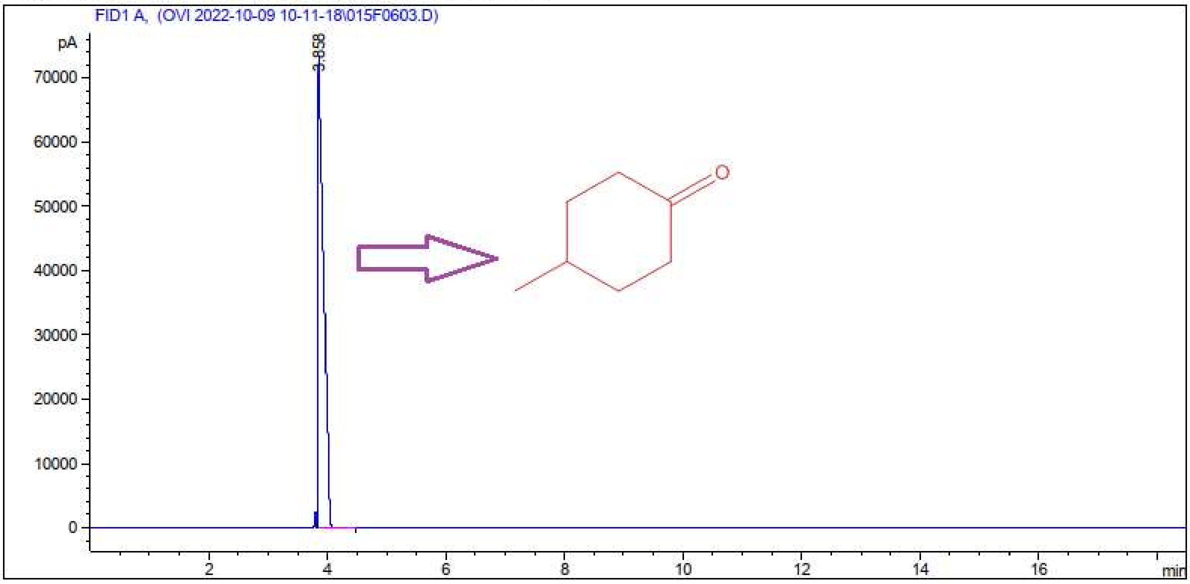


```
Signal 1: FID1 A,

Peak RetTime  Type   Width     Area      Area     Name
  #    [min]         [min]    [pA*s]       %
----|-------|------|-------|----------|--------|---------------------
   1   3.858 VB S    0.0977 4.21336e5  80.28265 ?
```

Catalyst	Substrate	Product	Catalyst ratio (mol%)	Retention time (RT) (min)	Area	Substrate conversion (%)
**Mixed MOF-Salinidol/Pd(II)**	4-methylcyclohexanol	4-methylcyclohexanone	0.3	3.858	421336	98

Figure S17. Chromatogram output for 4-methylcyclohexanone result of oxidation with Molecular Oxygen

```
Additional Info : Peak(s) manually integrated
```

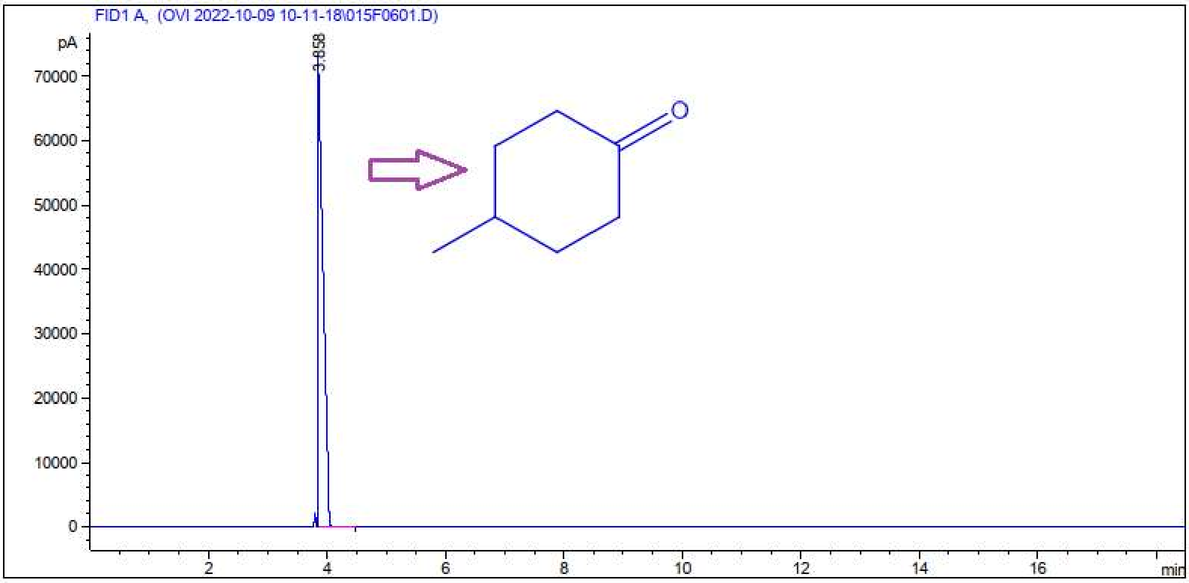


```
Signal 1: FID1 A,

Peak RetTime Type  Width     Area      Area    Name
  #   [min]        [min]   [pA*s]       %
----|-------|------|-------|----------|--------|---------------------
   1   3.858 VB S    0.0971 4.16944e5  80.23998 ?
```

Catalyst	Substrate	Product	Catalyst ratio (mol%)	Retention time (RT) (min)	Area	Substrate conversion (%)
**Mixed MOF-Salinidol/Pd(II)**	4-methylcyclohexanol	4-methylcyclohexanone	0.3	3.858	416944	97

Figure S18. Chromatogram output for 4-methylcyclohexanone result of oxidation with Air

Additional Info : Peak(s) manually integrated

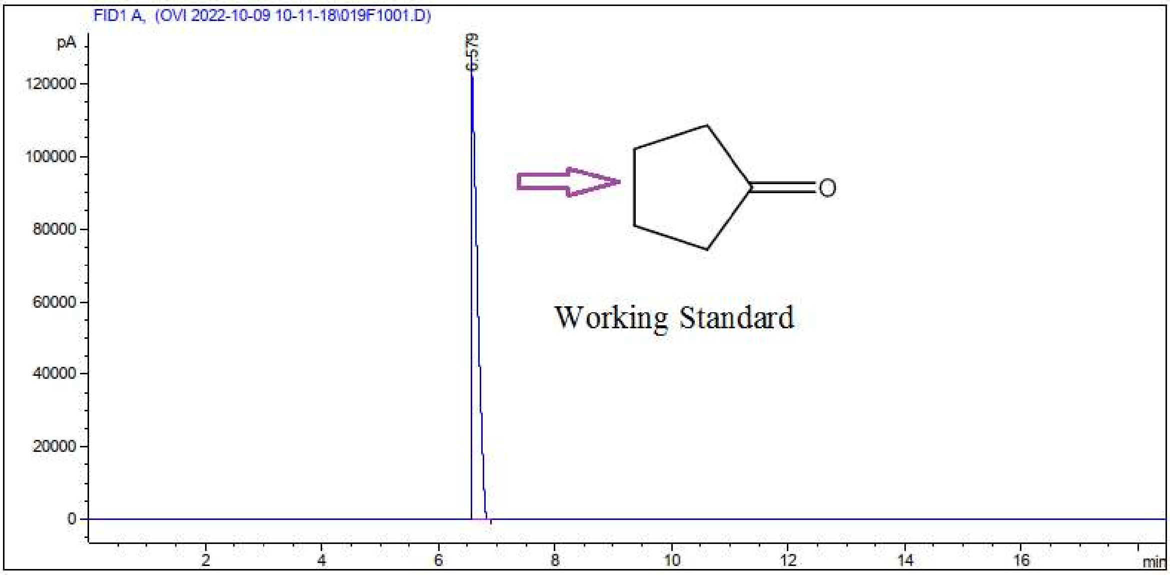

Signal 1: FID1 A,

```
Peak RetTime  Type   Width      Area       Area     Name
  #   [min]          [min]    [pA*s]        %
----|-------|------|-------|----------|--------|---------------------
   1   6.579 BB S    0.0829 8.50143e5  1.000e2
```

Catalyst	Substrate	Product	Catalyst ratio (mol%)	Retention time (RT) (min)	Area	Purity (%)
**NA**	NA	cyclopentanone	NA	6.579	850143	100

Figure S19. Chromatogram output for cyclopentanone working standard

Additional Info : Peak(s) manually integrated

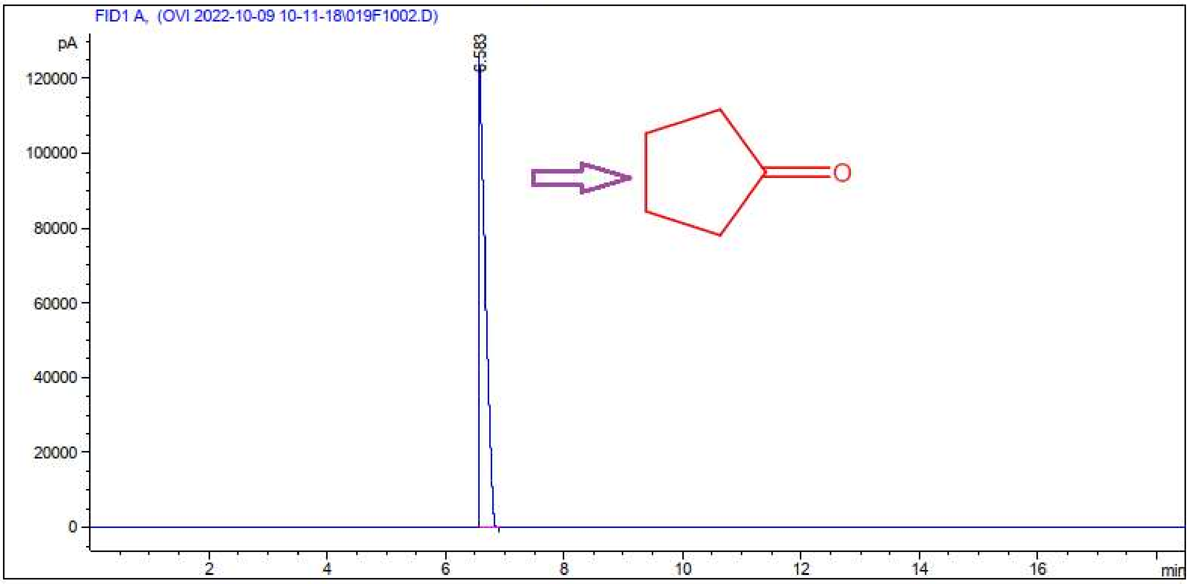


```
Signal 1: FID1 A,

Peak RetTime Type  Width     Area      Area    Name
  #   [min]        [min]   [pA*s]       %
----|-------|------|-------|----------|--------|---------------------
   1   6.583 BB S    0.0804 8.46372e5  1.000e2
```

Catalyst	Substrate	Product	Catalyst ratio (mol%)	Retention time (RT) (min)	Area	Substrate conversion (%)
**Mixed MOF-Salinidol/Pd(II)**	cyclopentanol	cyclopentanone	0.3	6.583	846372	99.5

Figure S20. Chromatogram output for cyclopentanone result of oxidation with Molecular Oxygen

Additional Info : Peak(s) manually integrated

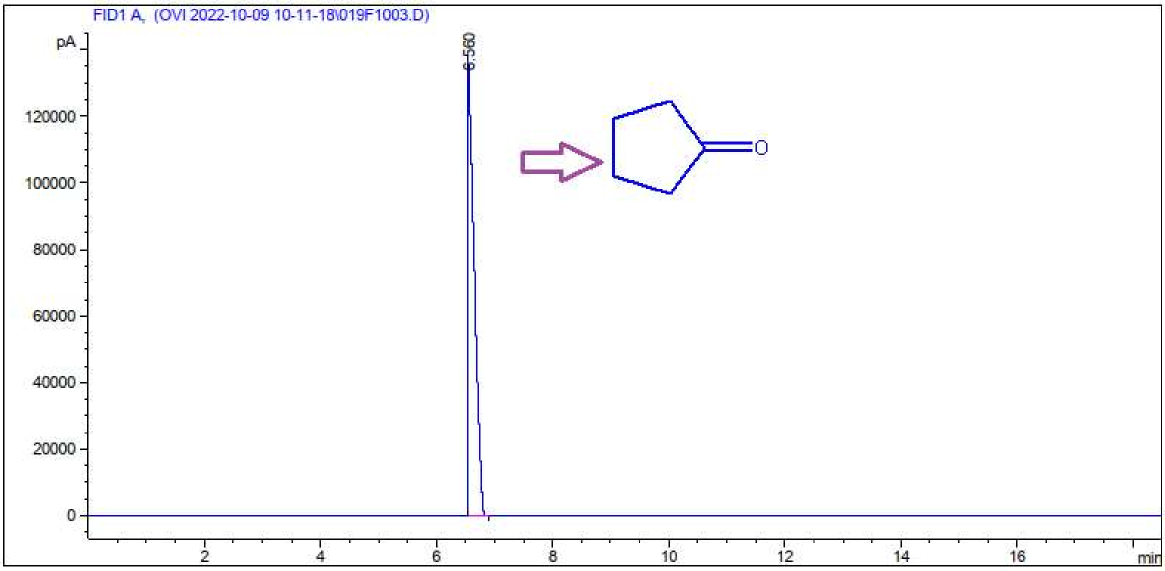

Signal 1: FID1 A,

Peak #	RetTime [min]	Type	Width [min]	Area [pA*s]	Area %	Name
1	6.560	BB S	0.0855	8.36431e5	1.000e2	

**Catalyst**	**Substrate**	**Product**	**Catalyst ratio (mol%)**	**Retention time (RT) (min)**	**Area**	**Substrate conversion (%)**
**Mixed MOF-Salinidol/Pd(II)**	cyclopentanol	cyclopentanone	0.3	6.560	836431	98.3

Figure S21. Chromatogram output for cyclopentanone result of oxidation with Air

```
Additional Info : Peak(s) manually integrated
```

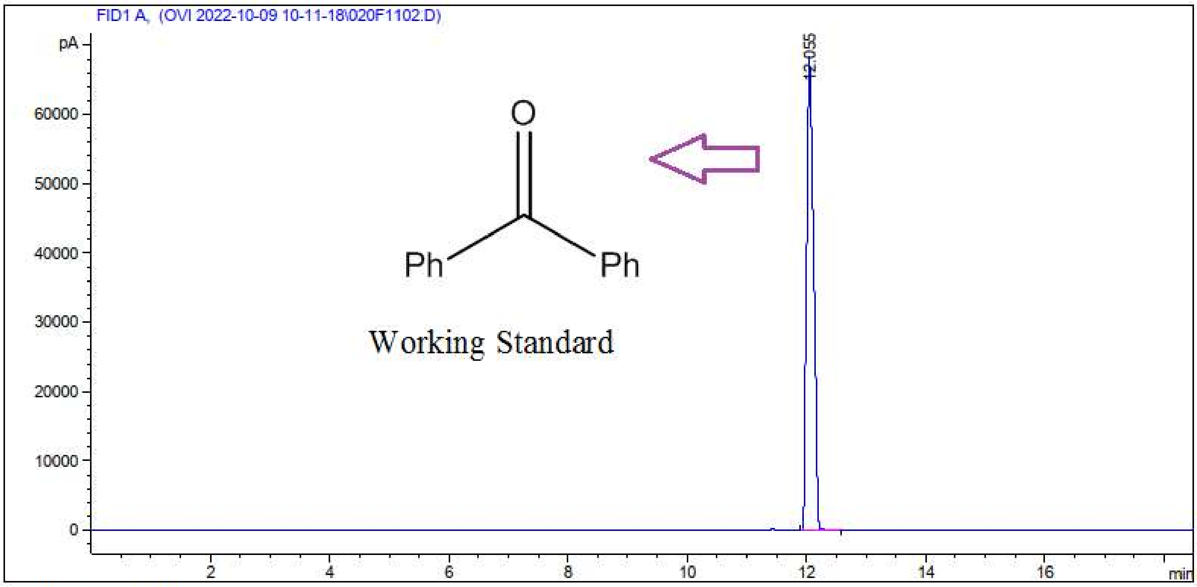


```
Signal 1: FID1 A,

Peak RetTime  Type   Width      Area      Area    Name
  #   [min]          [min]    [pA*s]       %
----|-------|------|-------|----------|--------|---------------------
   1  12.055 BB S    0.1085 5.51446e5  1.000e2  ?
```

Catalyst	Substrate	Product	Catalyst ratio (mol%)	Retention time (RT) (min)	Area	Purity(%)
**NA**	NA	benzophenone	NA	12.055	551446	100

Figure S22. Chromatogram output for benzophenone working standard

Additional Info : Peak(s) manually integrated

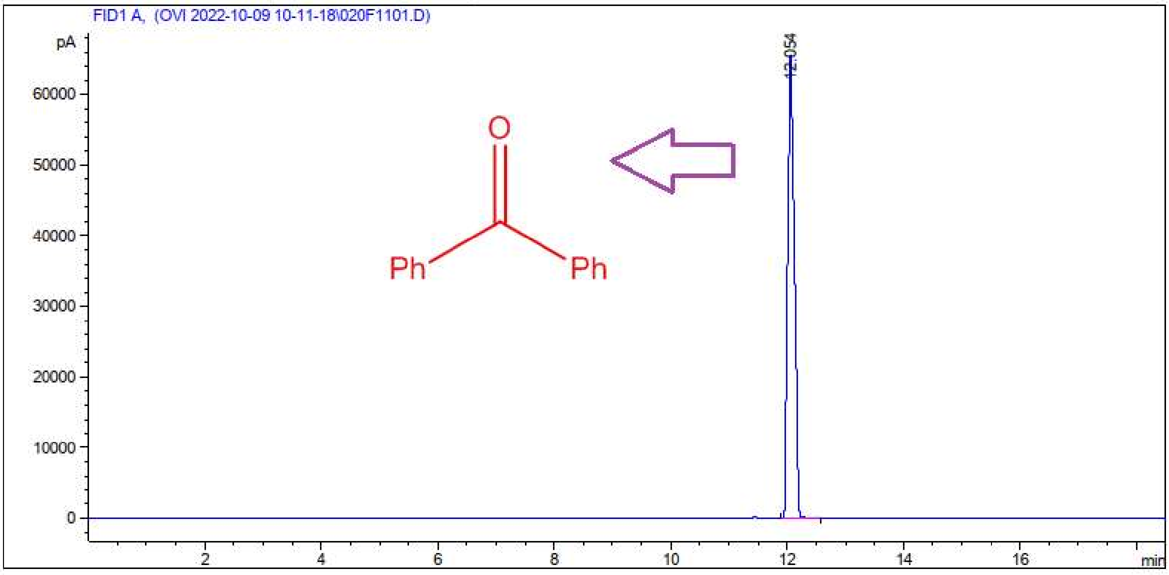


```
Signal 1: FID1 A,

Peak RetTime  Type   Width      Area       Area     Name
  #    [min]         [min]    [pA*s]        %
----|-------|------|-------|----------|--------|---------------------
   1  12.054 BB S    0.1032 5.00417e5  1.000e2  ?
```

Catalyst	Substrate	Product	Catalyst ratio (mol%)	Retention time (RT) (min)	Area	Substrate conversion (%)
**Mixed MOF-Salinidol/Pd(II)**	diphenylmethanol	benzophenone	20	12.054	500417	90.74

Figure S23. Chromatogram output for benzophenone result of oxidation with Molecular Oxygen

Additional Info : Peak(s) manually integrated

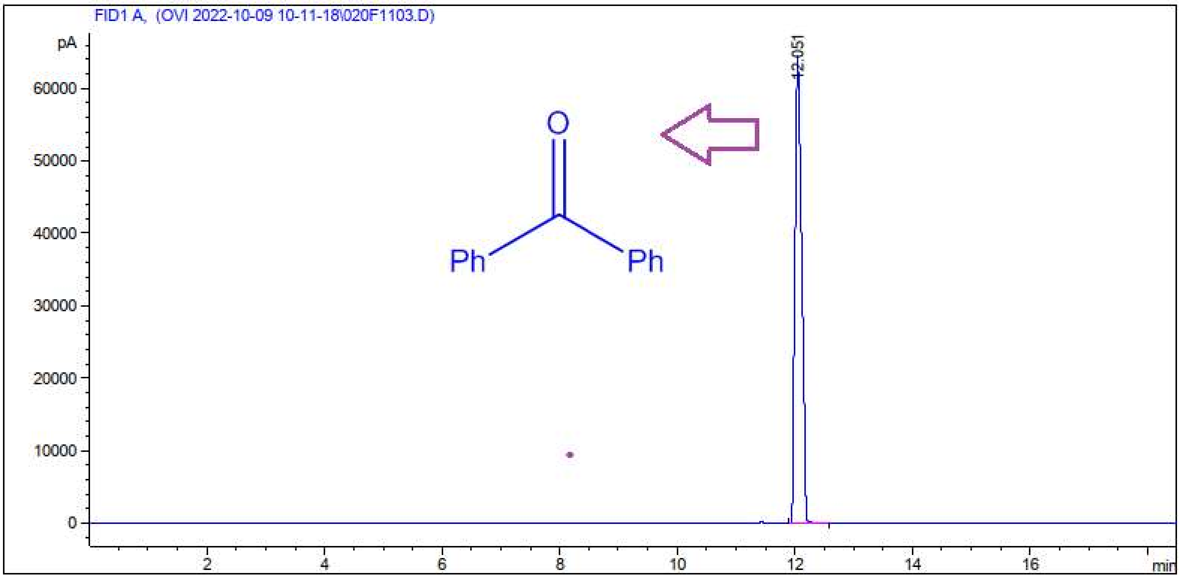


```
Signal 1: FID1 A,

Peak RetTime  Type   Width     Area       Area     Name
  #   [min]          [min]   [pA*s]         %
----|-------|------|-------|----------|--------|---------------------
   1  12.051 BB S    0.1041 4.94457e5  1.000e2  ?
```

Catalyst	Substrate	Product	Catalyst ratio (mol%)	Retention time (RT) (min)	Area	Substrate conversion (%)
**Mixed MOF-Salinidol/Pd(II)**	diphenylmethanol	benzophenone	0.3	12.051	494457	89.66

Figure S24. Chromatogram output for benzophenone result of oxidation with Air

```
Additional Info : Peak(s) manually integrated
```

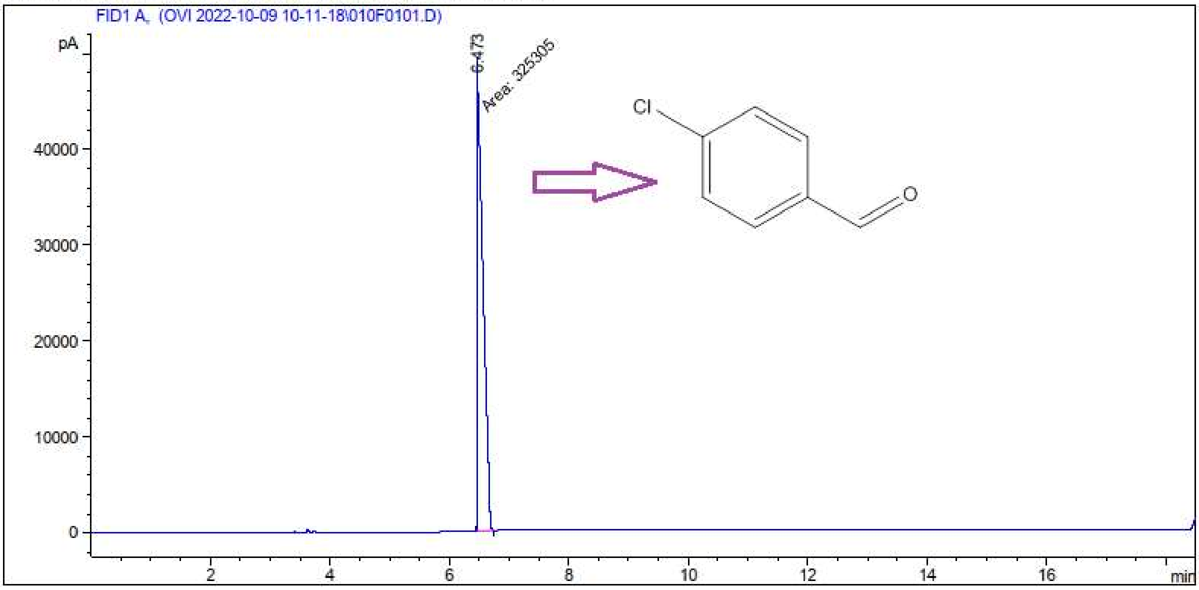


```
Peak RetTime Type  Width     Area      Height     Area
  #   [min]        [min]   [pA*s]       [pA]        %
----|-------|----|-------|----------|----------|--------|
   1   6.473 MM    0.1005 3.25305e5  5.39382e4  1.000e2
```

Catalyst	Substrate	Product	Catalyst ratio (mol%)	Retention time (RT) (min)	Area	Purity (%)
**NA**	NA	4-chlorobenzaldehyde	NA	6.473	325305	100

Figure S25. Chromatogram output for 4-chlorobenzaldehyde working standard

```
Additional Info : Peak(s) manually integrated
```

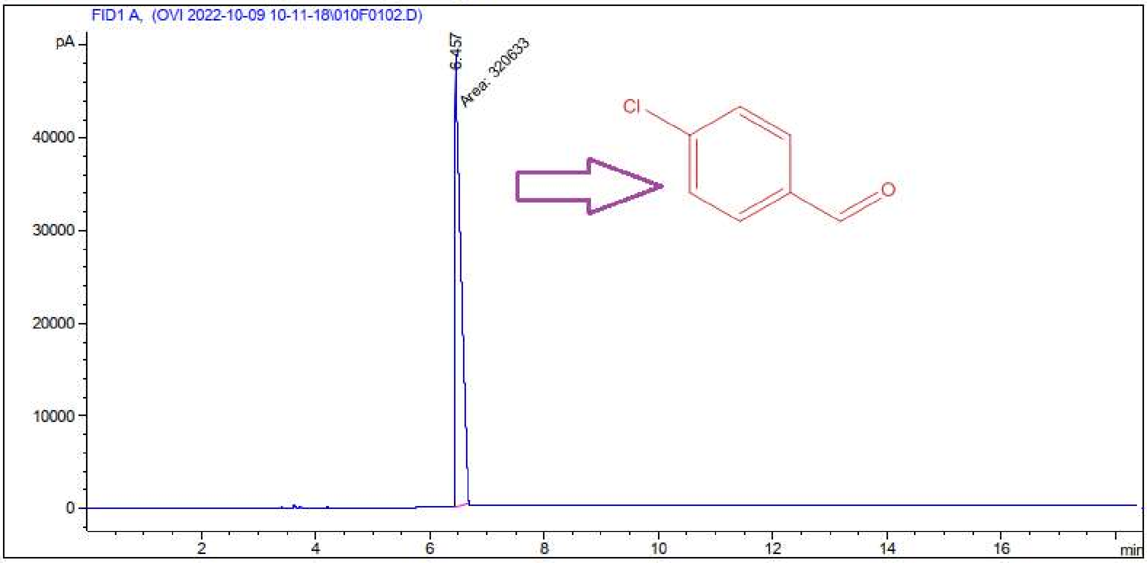


```
Peak RetTime Type  Width     Area      Height     Area
  #   [min]        [min]    [pA*s]      [pA]        %
----|-------|----|-------|----------|----------|--------|
   1   6.457 MM     0.0989 3.20633e5  5.40517e4  1.000e2
```

Catalyst	Substrate	Product	Catalyst ratio (mol%)	Retention time (RT) (min)	Area	Substrate conversion (%)
**Mixed MOF-Salinidol/Pd(II)**	(4-chlorophenyl)methanol	4-chlorobenzaldehyde	0.3	6.457	320633	98.5

Figure S26. Chromatogram output for 4-chlorobenzaldehyde result of oxidation with Molecular Oxygen

Additional Info : Peak(s) manually integrated

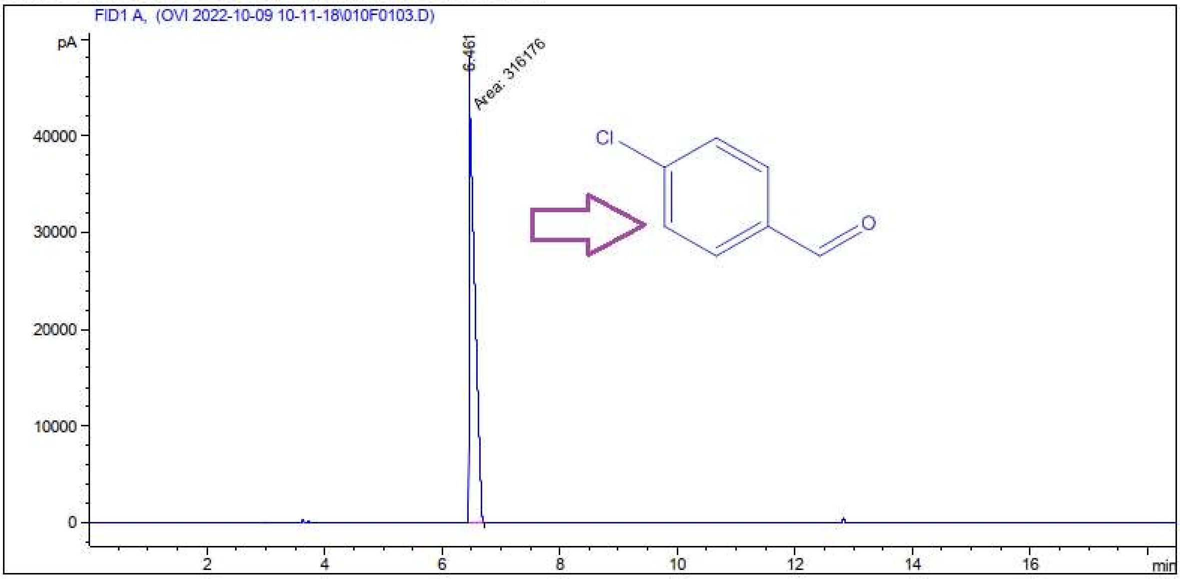

Peak #	RetTime [min]	Type	Width [min]	Area [pA*s]	Height [pA]	Area %
1	6.461	MM	0.1093	3.16176e5	4.82343e4	99.51105

Catalyst	Substrate	Product	Catalyst ratio (mol%)	Retention time (RT) (min)	Area	Substrate conversion (%)
**Mixed MOF-Salinidol/Pd(II)**	(4-chlorophenyl)methanol	4-chlorobenzaldehyde	0.3	6.461	316176	97.2

Figure S27. Chromatogram output for 4-chlorobenzaldehyde result of oxidation with Molecular Oxygen

Additional Info : Peak(s) manually integrated

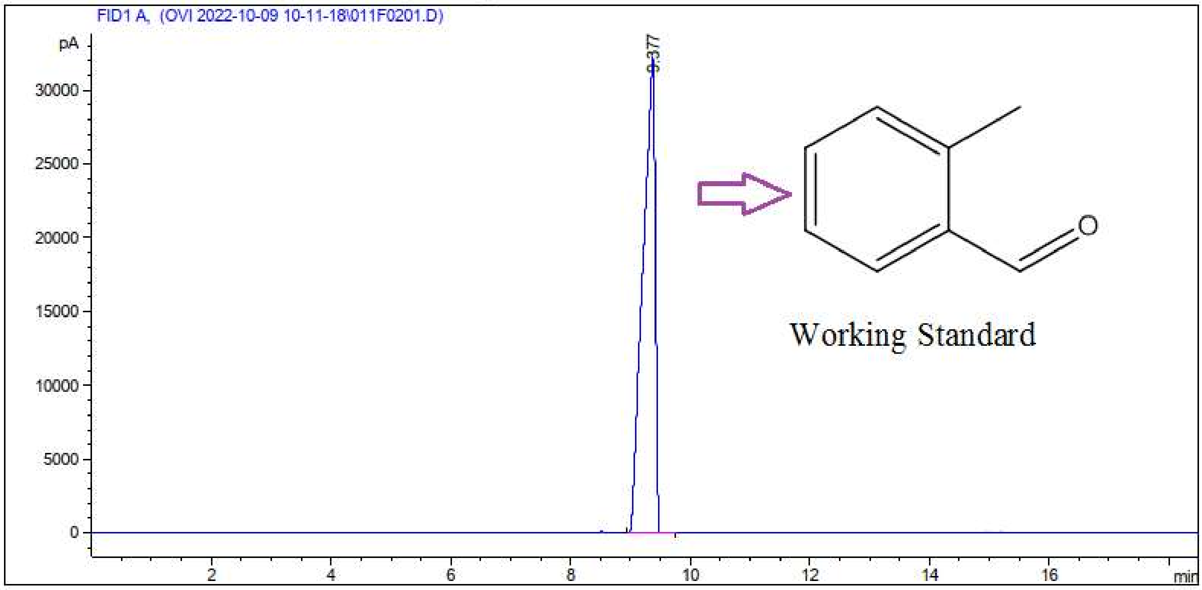


```
Peak RetTime Type  Width     Area      Height     Area
  #   [min]        [min]    [pA*s]      [pA]       %
----|-------|----|-------|----------|----------|--------|
   1   9.377 BB    0.1833 4.56081e5  3.21819e4  1.000e2
```

**Catalyst**	**Substrate**	**Product**	**Catalyst ratio (mol%)**	**Retention time (RT) (min)**	**Area**	**Purity (%)**
**NA**	NA	2-methylbenzaldehyde	NA	9.377	456081	100

Figure S28. Chromatogram output for 2-methylbenzaldehyde working standard

Additional Info : Peak(s) manually integrated

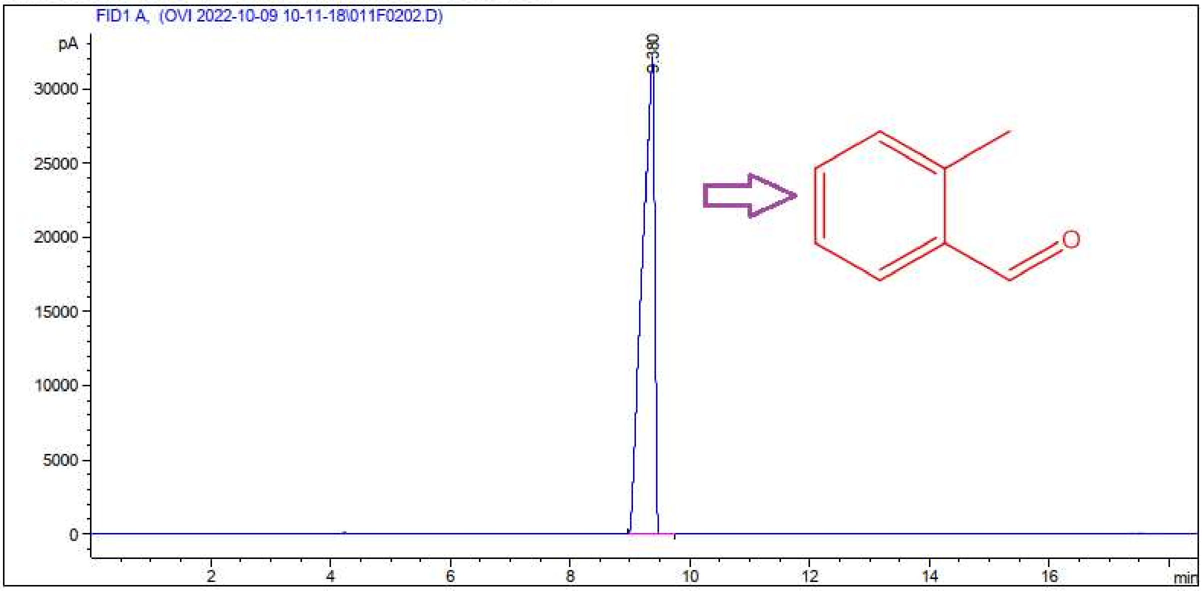

Peak #	RetTime [min]	Type	Width [min]	Area [pA*s]	Height [pA]	Area %
1	9.380	BB	0.1766	4.52097e5	3.20366e4	1.000e2

Catalyst	Substrate	Product	Catalyst ratio (mol%)	Retention time (RT) (min)	Area	Substrate conversion (%)
**Mixed MOF-Salinidol/Pd(II)**	o-tolylmethanol	2-methylbenzaldehyde	0.3	9.380	452097	99.1

Figure S29. Chromatogram output for 2-methylbenzaldehyde result of oxidation with Molecular Oxygen

Additional Info : Peak(s) manually integrated

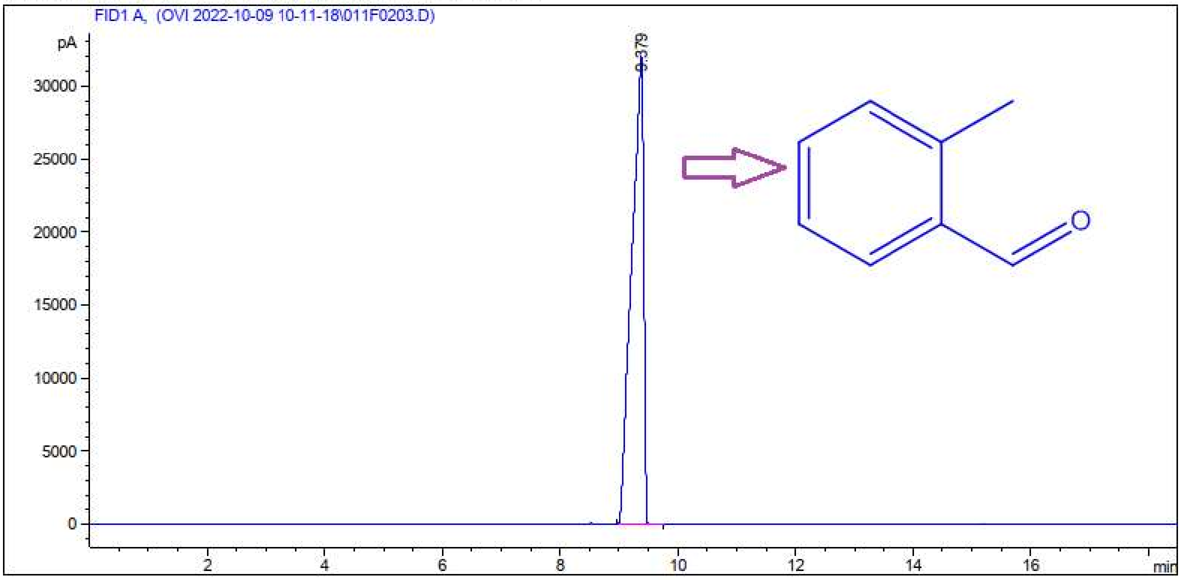

Peak #	RetTime [min]	Type	Width [min]	Area [pA*s]	Height [pA]	Area %
1	9.379	BB	0.1775	4.47955e5	3.19676e4	1.000e2

Catalyst	Substrate	Product	Catalyst ratio (mol%)	Retention time (RT) (min)	Area	Substrate conversion (%)
**Mixed MOF-Salinidol/Pd(II)**	o-tolylmethanol	2-methylbenzaldehyde	0.3	9.379	447955	98.2

Figure S30. Chromatogram output for 2-methylbenzaldehyde result of oxidation with Air

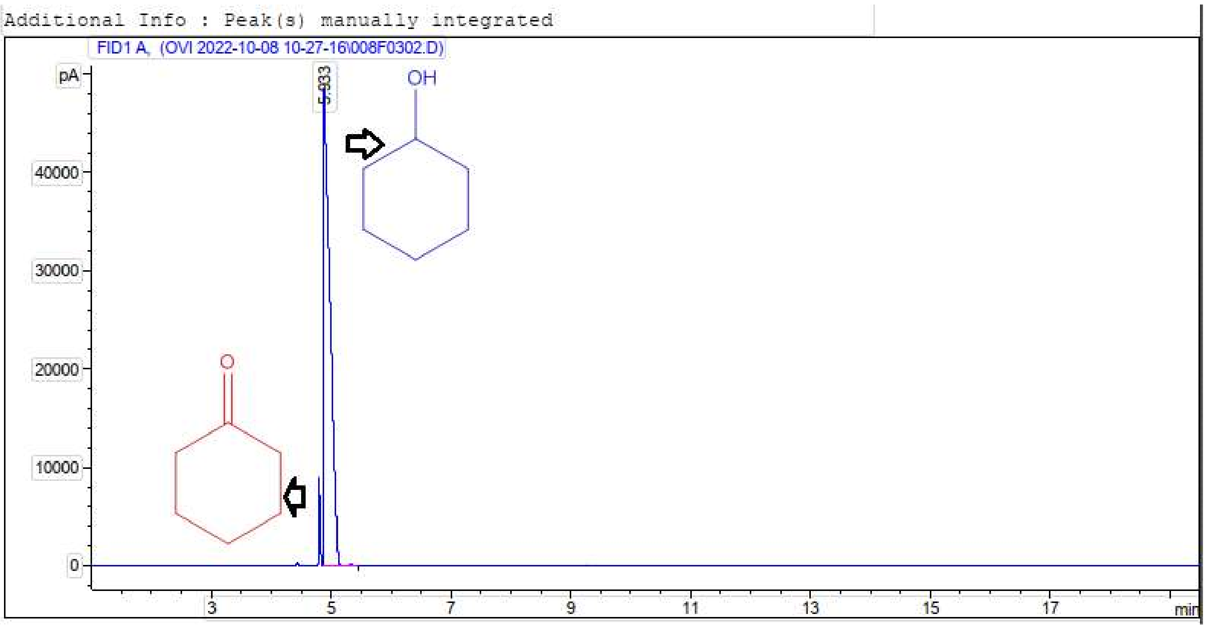

Peak #	RetTime [min]	Type	Width [min]	Area [pA*s]	Area %	Name
1	5.325		0.0000	0.21685	0.59900	
2	5.933	VB S	0.1184	3.39737e5	0.941e2	?

Totals :				3.39737e5		

Catalyst	Substrate	Product	Catalyst ratio (mol%)	Retention time (min)	Area	Substrate conversion (%)
**Salinidol/Pd(II)**	cyclohexanone	cyclohexanol	0.3	5.933		5.99

Figure S31. Chromatogram output for cyclohexanone result of result of oxidation with Air catalyzed by Salinidol/Pd(II)

3. The results of catalytic activity for hydrogenation reaction for nitrobenzene and derivatives

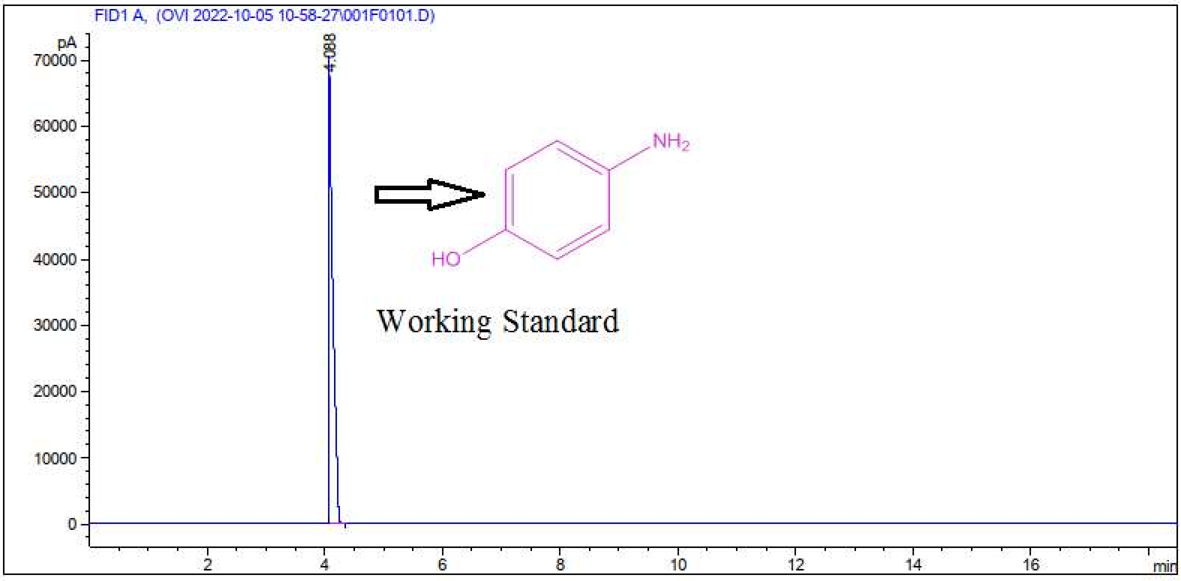


```
Signal 1: FID1 A,

Peak RetTime Type  Width     Area      Height     Area
  #   [min]        [min]   [pA*s]      [pA]        %
----|-------|----|-------|----------|----------|--------|
   1   4.088 BB S  0.0549 2.97638e5  6.81636e4  1.000e2
```

Catalyst	Substrate	Product	Catalyst ratio (mol%)	Retention time (RT) (min)	Area	Purity (%)
**NA**	NA	4-aminophenol	NA	4.088	297638	100

Figure S32**.** Chromatogram output for 4-aminophenol working standard

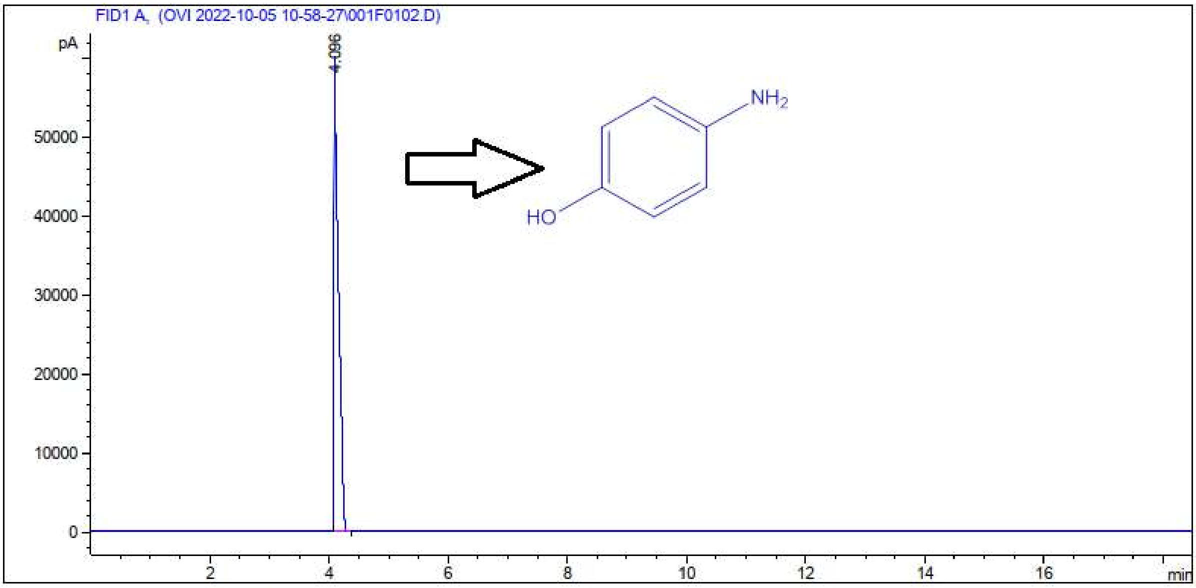

Peak #	RetTime [min]	Type	Width [min]	Area [pA*s]	Height [pA]	Area %
1	4.096	BB S	0.0622	2.93192e5	5.97854e4	1.000e2

Catalyst	Substrate	Product	Catalyst ratio (mol%)	Retention time (RT) (min)	Area	Substrate conversion (%)
**Mixed MOF-Salinidol/Pd(II)**	4-nitrophenol	4-aminophenol	0.1	4.096	293192	98.5

Figure S33. Chromatogram output for 2-methylbenzaldehyde result of hydrogenation reaction for nitrobenzene and derivatives

Additional Info : Peak(s) manually integrated

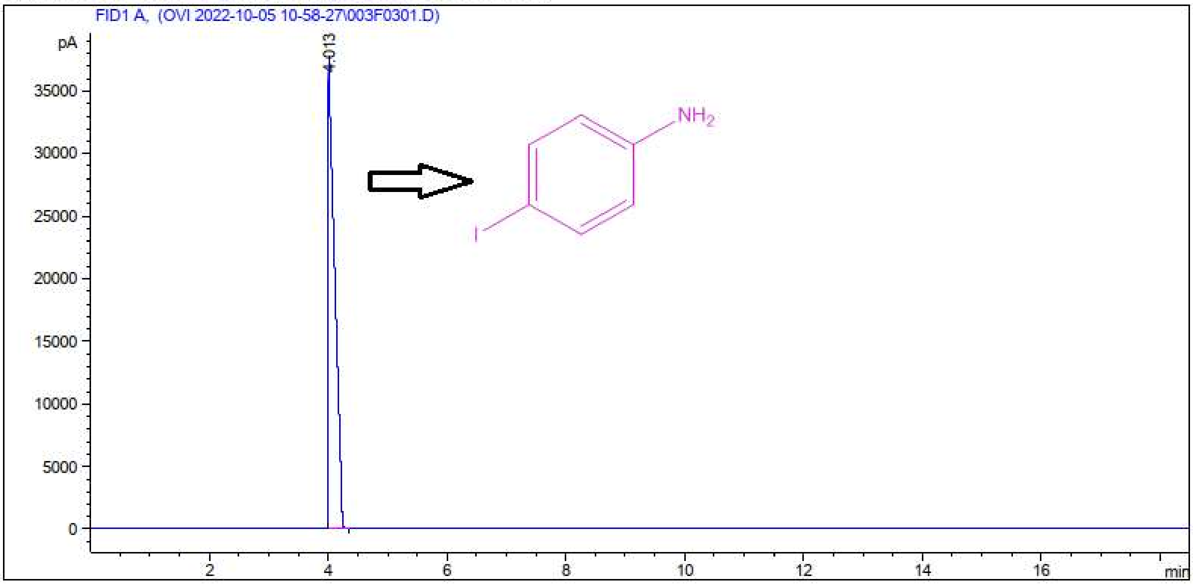


```
Signal 1: FID1 A,

Peak RetTime Type  Width     Area      Height     Area
  #   [min]        [min]    [pA*s]      [pA]       %
----|-------|----|-------|----------|----------|--------|
   1   4.013 BB S  0.0889 2.76067e5  3.79526e4  1.000e2
```

Catalyst	Substrate	Product	Catalyst ratio (mol%)	Retention time (RT) (min)	Area	Purity (%)
**NA**	NA	4-iodoaniline	NA	4.013	276067	100

Figure S34. Chromatogram output for 4-iodoaniline working standard

```
Additional Info : Peak(s) manually integrated
```

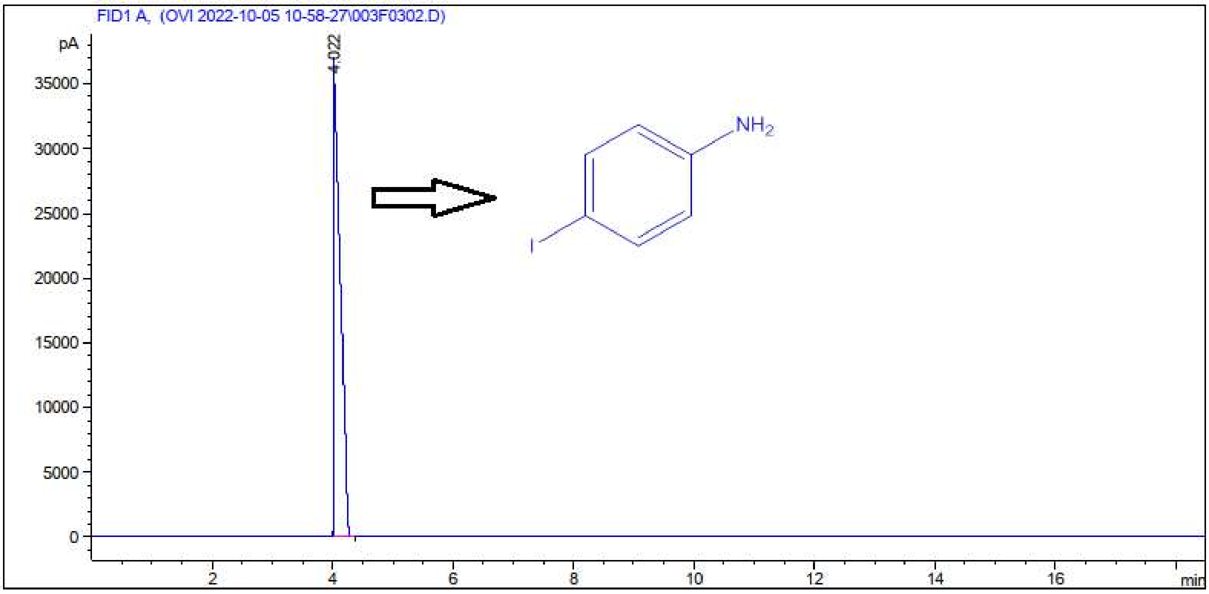


```
Signal 1: FID1 A,

Peak RetTime Type  Width     Area      Height     Area
  #   [min]        [min]    [pA*s]      [pA]       %
----|-------|----|-------|----------|----------|--------|
   1   4.022 BB S  0.0930 2.73758e5  3.67293e4  1.000e2
```

Catalyst	Substrate	Product	Catalyst ratio (mol%)	Retention time (RT) (min)	Area	Substrate conversion (%)
**Mixed MOF-Salinidol/Pd(II)**	1-iodo-4-nitrobenzene	4-iodoaniline	0.1	4.022	273758	99.1

Figure S35. Chromatogram output for 4-iodoaniline result of hydrogenation reaction for nitrobenzene and derivatives

Additional Info : Peak(s) manually integrated

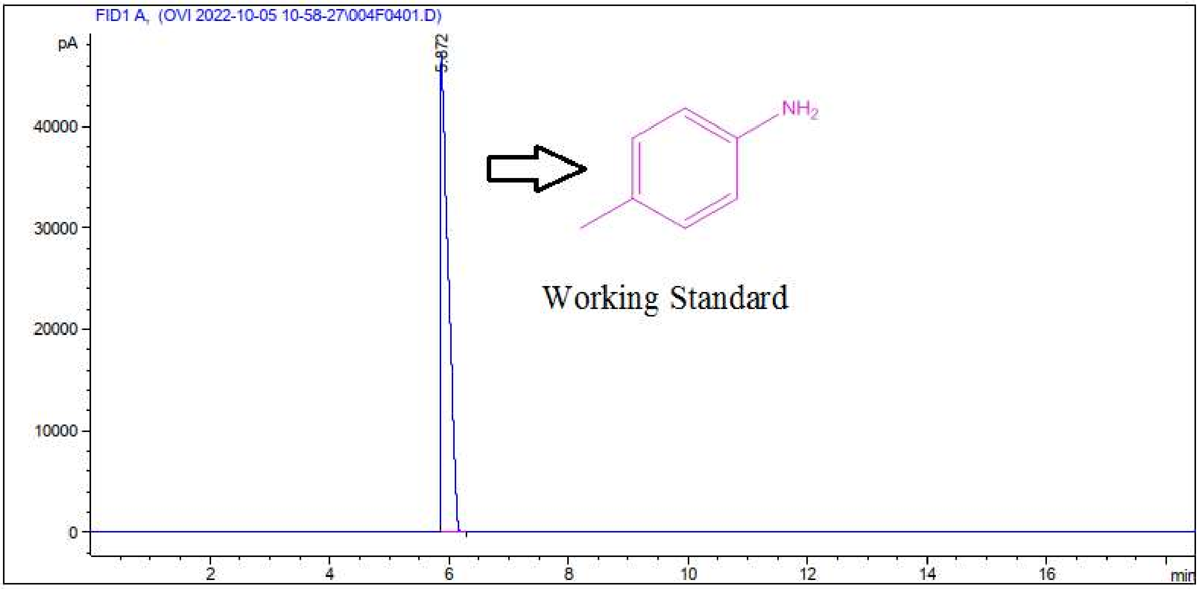


```
Signal 1: FID1 A,

Peak RetTime Type  Width     Area      Height     Area
  #   [min]        [min]    [pA*s]      [pA]        %
----|-------|----|-------|----------|----------|--------|
   1   5.872 BB S  0.1076 4.05134e5  4.65234e4  1.000e2
```

Catalyst	Substrate	Product	Catalyst ratio (mol%)	Retention time (RT) (min)	Area	Purity (%)
**NA**	NA	p-toluidine	NA	5.872	405134	100

Figure S36. Chromatogram output for p-toluidine working standard

Additional Info : Peak(s) manually integrated

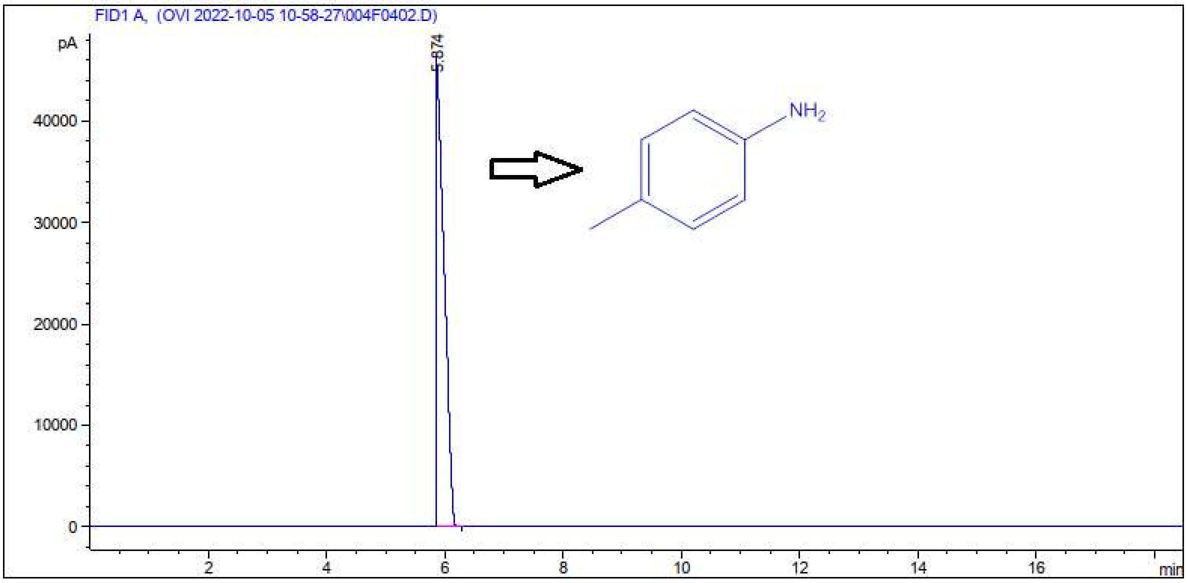

Signal 1: FID1 A,

Peak #	RetTime [min]	Type	Width [min]	Area [pA*s]	Height [pA]	Area %
1	5.874	BB S	0.1054	4.00146e5	4.55741e4	1.000e2

Catalyst	Substrate	Product	Catalyst ratio (mol%)	Retention time (RT) (min)	Area	Substrate conversion (%)
**Mixed MOF-Salinidol/Pd(II)**	1-methyl-4-nitrobenzene	p-toluidine	0.1	5.874	400146	98.7

Figure S37. Chromatogram output for p-toluidine result of hydrogenation reaction for nitrobenzene and derivatives

Additional Info : Peak(s) manually integrated

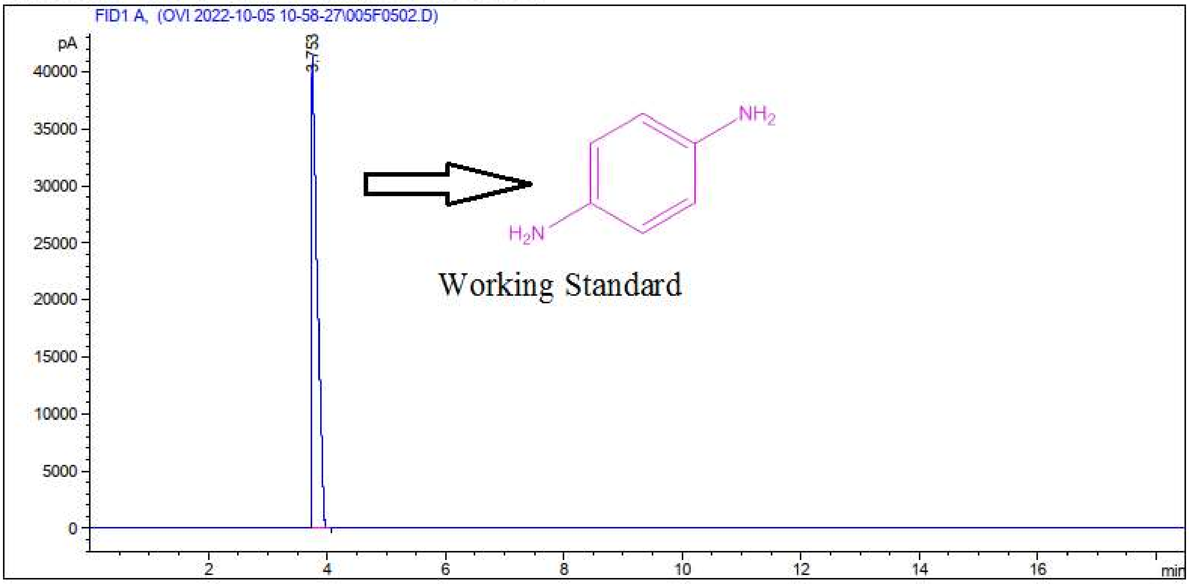


```
Signal 1: FID1 A,

Peak RetTime Type  Width     Area      Height     Area
  #    [min]        [min]    [pA*s]     [pA]        %
----|-------|----|-------|----------|----------|--------|
   1   3.753 BB S  0.0775 2.46429e5  4.08071e4  1.000e2
```

Catalyst	Substrate	Product	Catalyst ratio (mol%)	Retention time (RT) (min)	Area	Purity (%)
**NA**	NA	benzene-1,4-diamine	NA	3.753	246429	100

Figure S38. Chromatogram output for benzene-1,4-diamine working standard

Additional Info : Peak(s) manually integrated

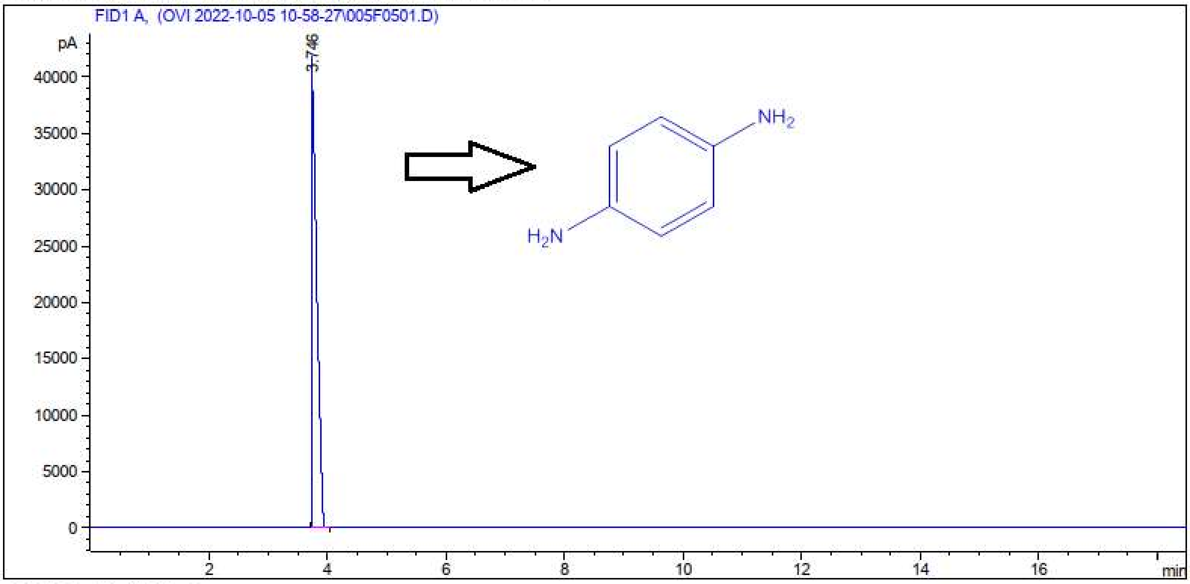

Signal 1: FID1 A,

```
Peak RetTime Type  Width     Area      Height     Area
  #   [min]        [min]   [pA*s]       [pA]        %
----|-------|----|-------|----------|----------|--------|
   1   3.746 BB S  0.0735 2.44698e5  4.10758e4  1.000e2
```

**Catalyst**	**Substrate**	**Product**	**Catalyst ratio (mol%)**	**Retention time (RT) (min)**	**Area**	**Substrate conversion (%)**
**Mixed MOF-Salinidol/Pd(II)**	4-nitroaniline	benzene-1,4-diamine	0.1	3.746	244698	99.2

Figure S39. Chromatogram output for benzene-1,4-diamine result of hydrogenation reaction for nitrobenzene and derivatives

Additional Info : Peak(s) manually integrated

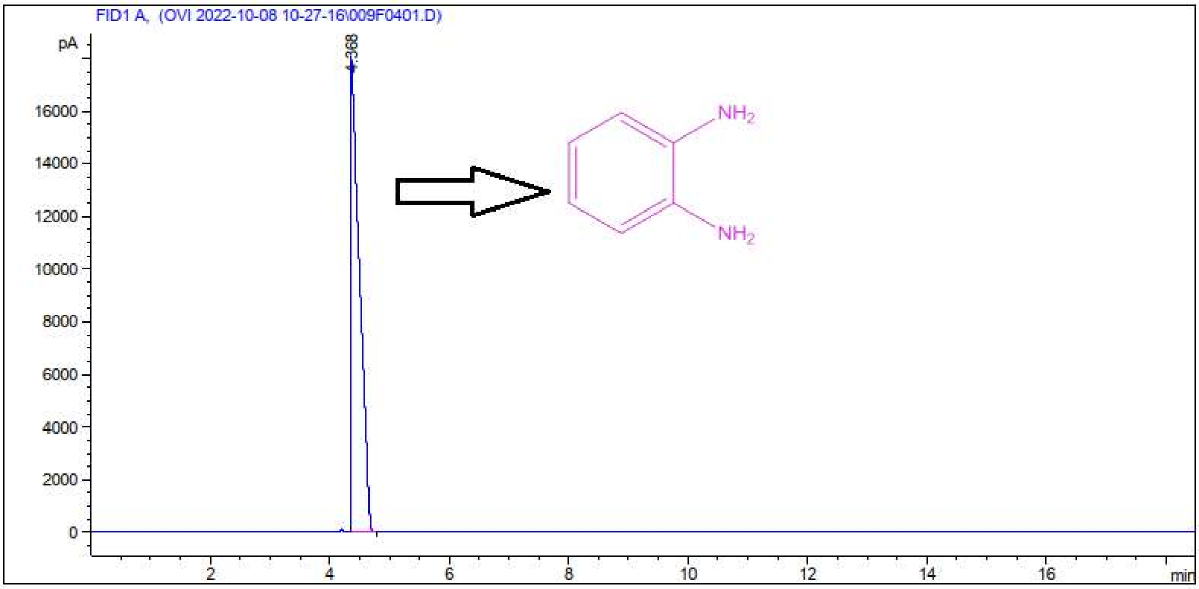


```
Signal 1: FID1 A,

Peak RetTime  Type   Width     Area      Area    Name
  #   [min]          [min]   [pA*s]       %
----|-------|------|-------|----------|--------|---------------------
   1   4.368 BB S    0.1192 1.81934e5  1.000e2  ?
```

Catalyst	Substrate	Product	Catalyst ratio (mol%)	Retention time (RT) (min)	Area	Purity (%)
**NA**	NA	benzene-1,2-diamine	NA	4.368	181934	100

Figure S40. Chromatogram output for benzene-1,2-diamine working standard

Additional Info : Peak(s) manually integrated

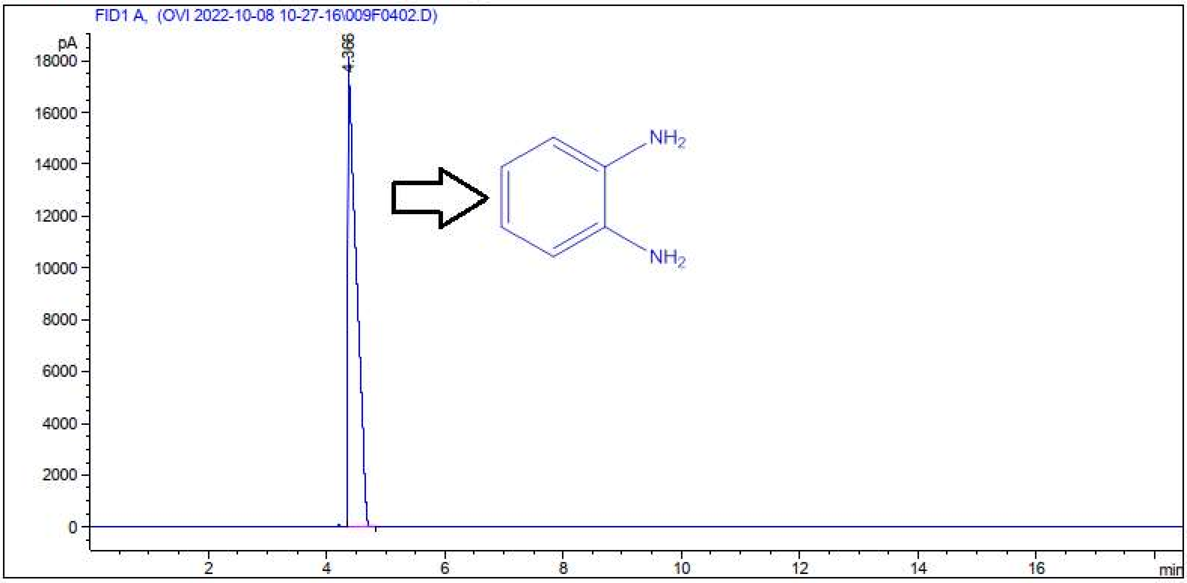

Signal 1: FID1 A,

```
Peak RetTime  Type   Width     Area      Area     Name
  #   [min]          [min]   [pA*s]       %
----|-------|------|-------|----------|--------|---------------------
   1   4.366 BB S    0.1218 1.76767e5  1.000e2  ?
```

Catalyst	Substrate	Product	Catalyst ratio (mol%)	Retention time (RT) (min)	Area	Substrate conversion (%)
**Mixed MOF-Salinidol/Pd(II)**	2-nitroaniline	benzene-1,2-diamine	0.1	4.366	176767	97.1

Figure S41. Chromatogram output for benzene-1,2-diamine result of hydrogenation reaction for nitrobenzene and derivatives

Additional Info : Peak(s) manually integrated

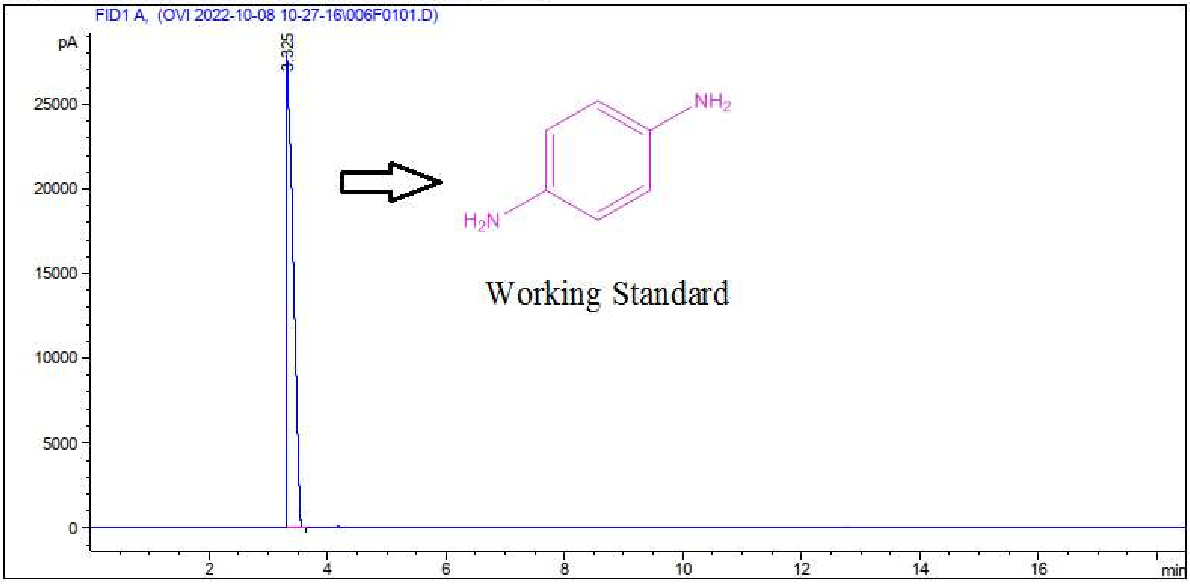


```
Signal 1: FID1 A,

Peak RetTime  Type  Width     Area      Area    Name
  #   [min]         [min]    [pA*s]       %
----|-------|------|-------|----------|--------|---------------------
   1   3.325 BB S    0.0905 2.07236e5  1.000e2
```

Catalyst	Substrate	Product	Catalyst ratio (mol%)	Retention time (RT) (min)	Area	Purity (%)
**NA**	NA	benzene-1,4-diamine	NA	3.325	207236	100

Figure S42**.** Chromatogram output for benzene-1,4-diamine working standard

Additional Info : Peak(s) manually integrated

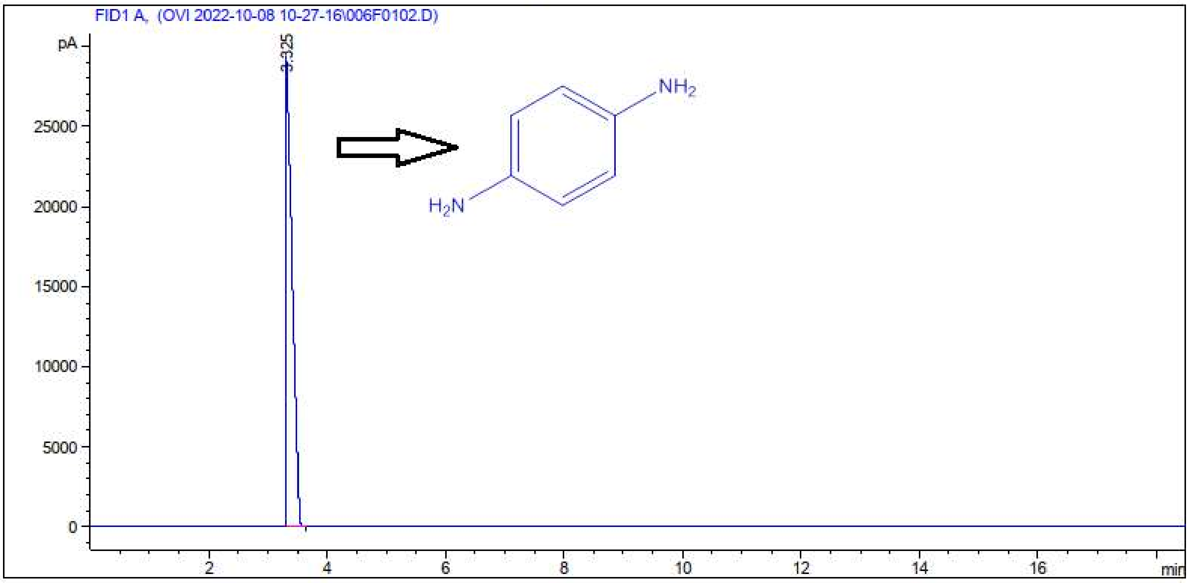


```
Signal 1: FID1 A,

Peak RetTime  Type   Width     Area      Area    Name
  #   [min]          [min]   [pA*s]        %
----|-------|------|-------|----------|--------|---------------------
   1   3.325 BB S    0.0851 1.98221e5  1.000e2
```

Catalyst	Substrate	Product	Catalyst ratio (mol%)	Retention time (RT) (min)	Area	Substrate conversion (%)
**Mixed MOF-Salinidol/Pd(II)**	1,4-dinitrobenzene	benzene-1,4-diamine	0.1	3.325	198221	95.6

Figure S43**.** Chromatogram output for benzene-1,4-diamine result of hydrogenation reaction for nitrobenzene and derivatives

```
Additional Info : Peak(s) manually integrated
```

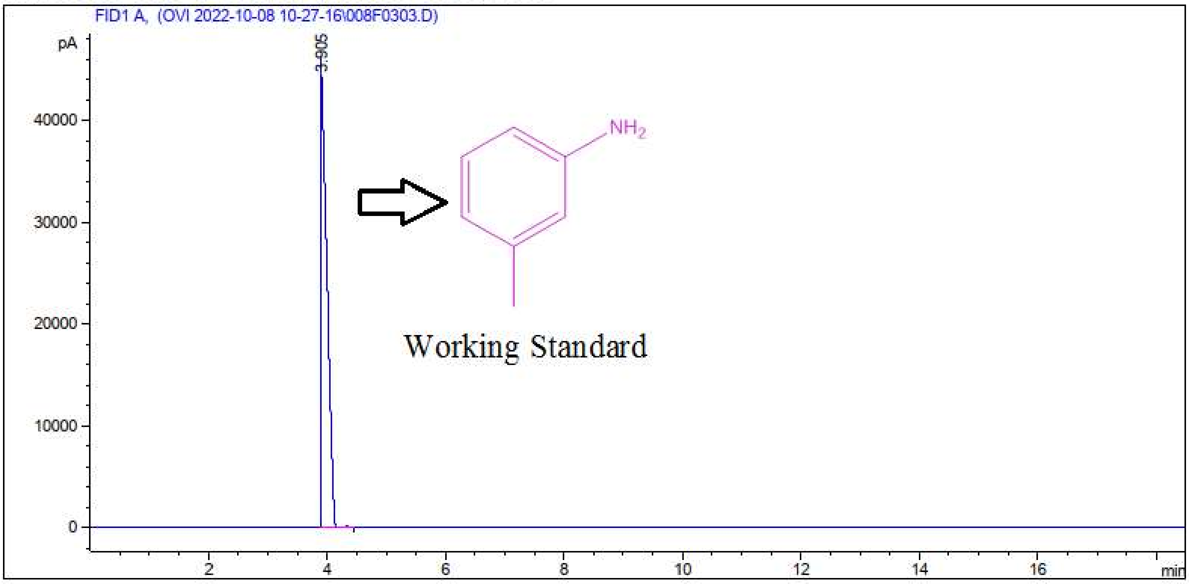


```
Signal 1: FID1 A,

Peak RetTime  Type   Width     Area      Area    Name
  #   [min]          [min]   [pA*s]       %
----|-------|------|-------|----------|--------|---------------------
   1   3.905 VB S    0.1164 3.16650e5  1.000e2
```

Catalyst	Substrate	Product	Catalyst ratio (mol%)	Retention time (RT) (min)	Area	Purity (%)
**NA**	NA	m-toluidine	NA	3.905	316650	100

Figure S44. Chromatogram output for m-toluidine working standard

Additional Info : Peak(s) manually integrated

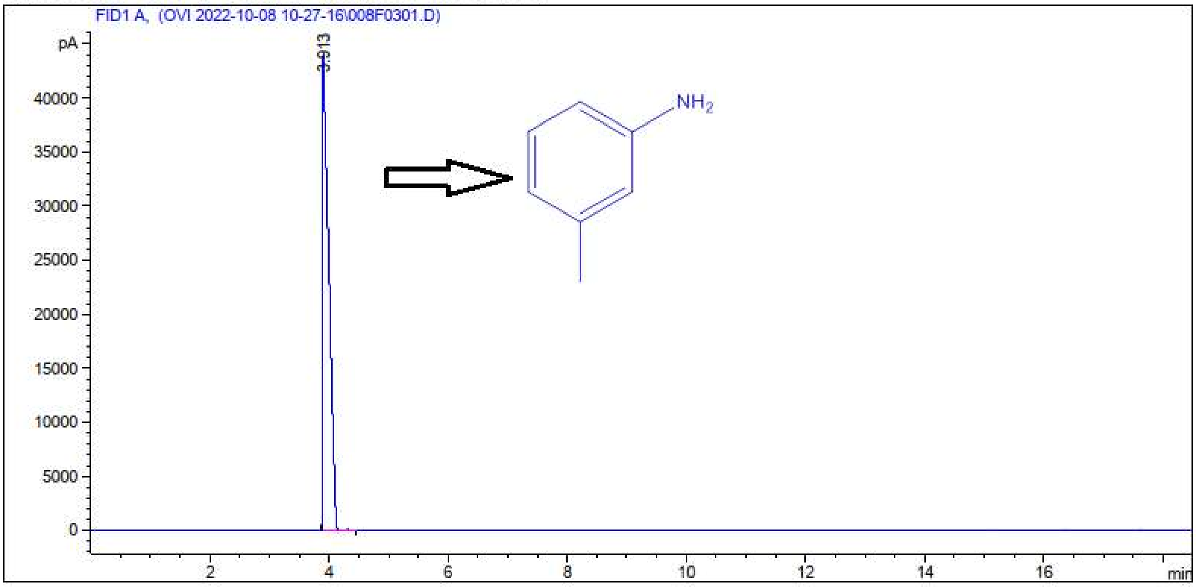

Signal 1: FID1 A,

```
Peak RetTime  Type   Width     Area      Area     Name
  #   [min]          [min]   [pA*s]       %
----|-------|------|-------|----------|--------|---------------------
   1   3.913 VB S    0.1061 3.06187e5  1.000e2  ?
```

Catalyst	Substrate	Product	Catalyst ratio (mol%)	Retention time (RT) (min)	Area	Substrate conversion (%)
**Mixed MOF-Salinidol/Pd(II)**	1-methyl-3-nitrobenzene	m-toluidine	0.1	3.913	306187	96.6

Figure S45. Chromatogram output for m-toluidine result of hydrogenation reaction for nitrobenzene and derivatives

Additional Info : Peak(s) manually integrated

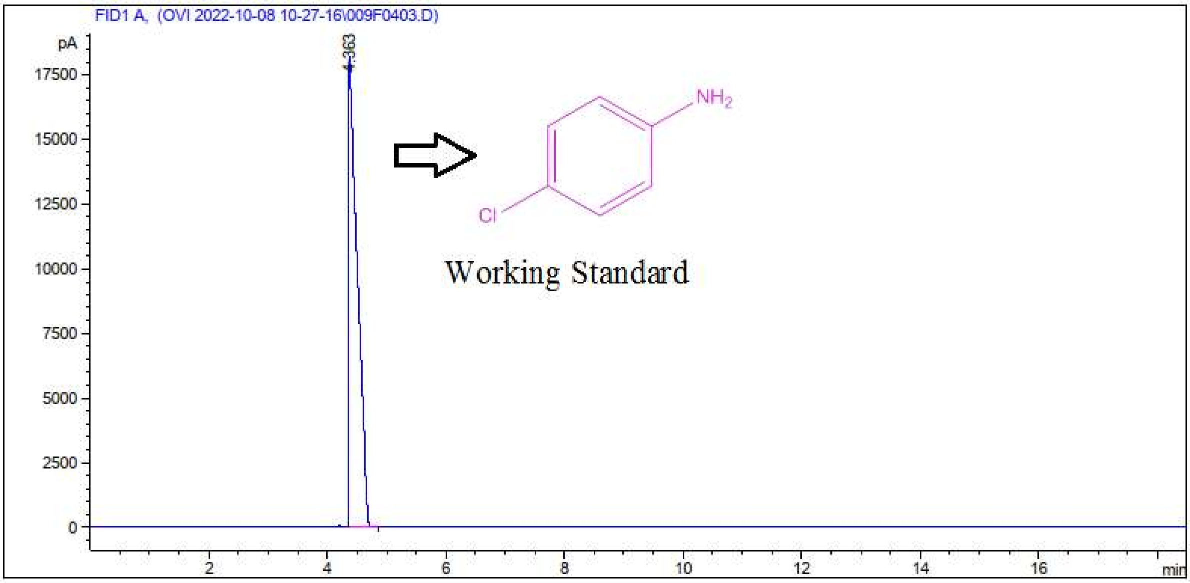


```
Signal 1: FID1 A,

Peak RetTime  Type   Width     Area      Area    Name
  #   [min]          [min]    [pA*s]      %
----|-------|------|-------|----------|--------|---------------------
   1   4.363 BB S    0.1169 1.88851e5  1.000e2  ?
```

Catalyst	Substrate	Product	Catalyst ratio (mol%)	Retention time (RT) (min)	Area	Purity (%)
**NA**	NA	4-chloroaniline	NA	4.363	188851	100

Figure S46. Chromatogram output for 4-chloroaniline working standard

Additional Info : Peak(s) manually integrated

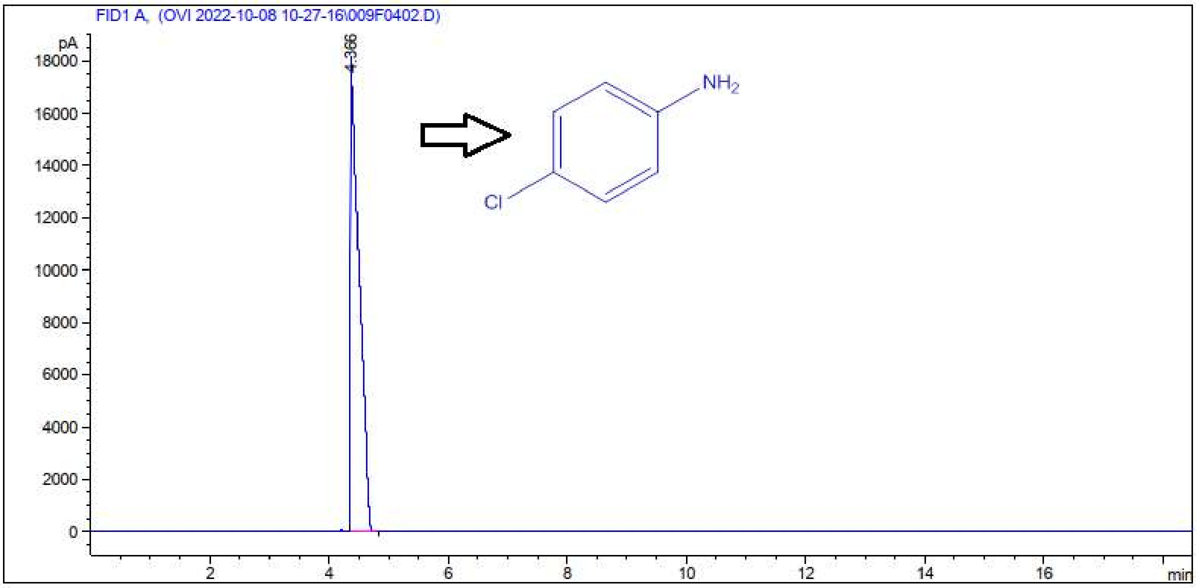

Signal 1: FID1 A,

Peak #	RetTime [min]	Type	Width [min]	Area [pA*s]	Area %	Name
1	4.366	BB S	0.1218	1.65251e5	1.000e2	?

Catalyst	Substrate	Product	Catalyst ratio (mol%)	Retention time (RT) (min)	Area	Substrate conversion (%)
**Mixed MOF-Salinidol/Pd(II)**	1-chloro-4-nitrobenzene	4-chloroaniline	0.1	4.366	165251	87.5

Figure S47. Chromatogram output for 4-chloroaniline result of hydrogenation reaction for nitrobenzene and derivatives

```
Additional Info : Peak(s) manually integrated
```

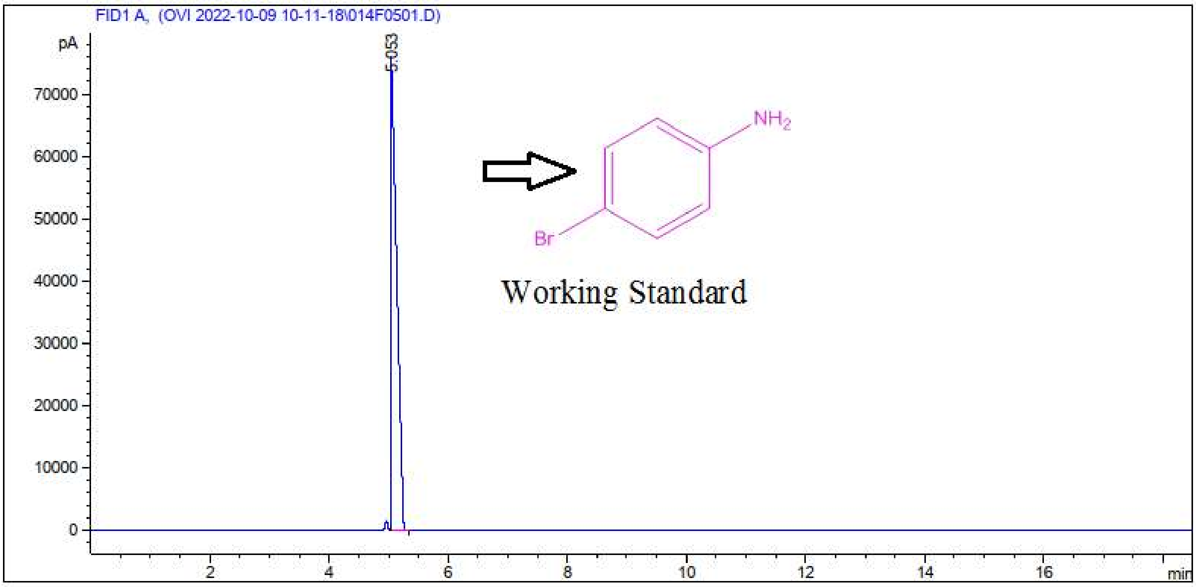


```
Signal 1: FID1 A,

Peak RetTime  Type   Width     Area      Area    Name
  #   [min]          [min]    [pA*s]      %
----|-------|------|-------|----------|--------|---------------------
   1   5.863 VB S    0.0505 1.45740e4   2.81248 ?
```

**Catalyst**	**Substrate**	**Product**	**Catalyst ratio (mol%)**	**Retention time (RT) (min)**	**Area**	**Purity (%)**
**NA**	NA	4-bromoaniline	NA	5.863	145740	100

Figure S48**.** Chromatogram output for 4-bromoaniline working standard

Additional Info : Peak(s) manually integrated

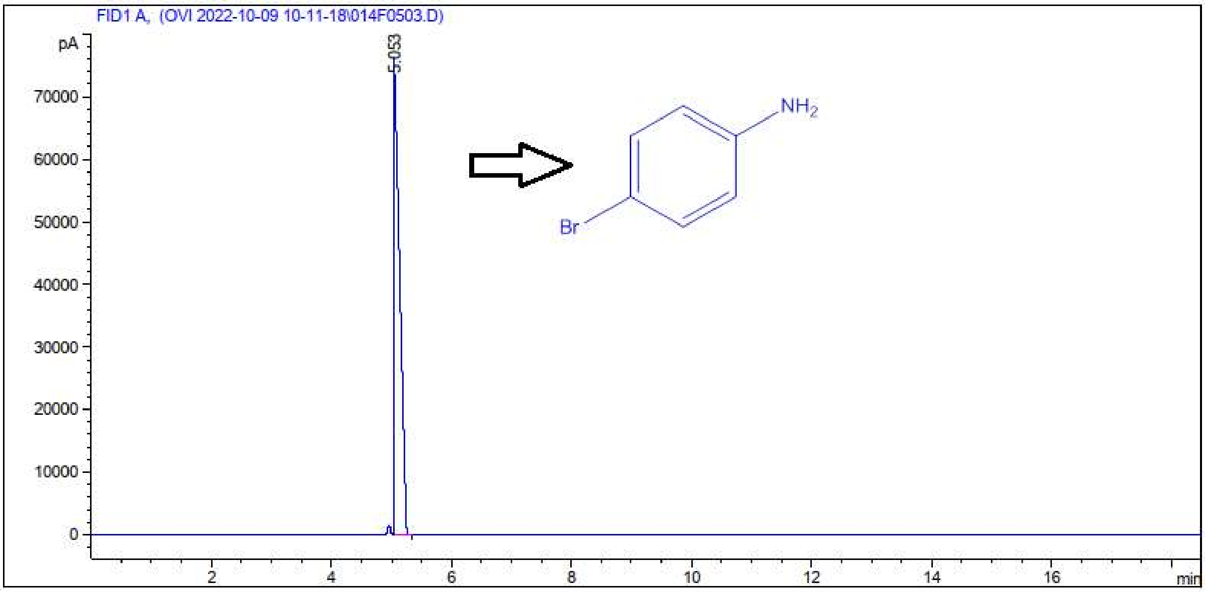

Signal 1: FID1 A,

```
Peak RetTime  Type   Width     Area      Area    Name
  #   [min]          [min]    [pA*s]      %
----|-------|------|-------|----------|--------|---------------------
   1   5.863 VB S    0.0501 1.33214e4   2.80834 ?
```

Catalyst	Substrate	Product	Catalyst ratio (mol%)	Retention time (RT) (min)	Area	Substrate conversion (%)
**Mixed MOF-Salinidol/Pd(II)**	1-bromo-4-nitrobenzene	4-bromoaniline	0.1	5.863	133214	91.4

Figure S49. Chromatogram output for 4-bromoaniline result of hydrogenation reaction for nitrobenzene and derivatives

Additional Info : Peak(s) manually integrated

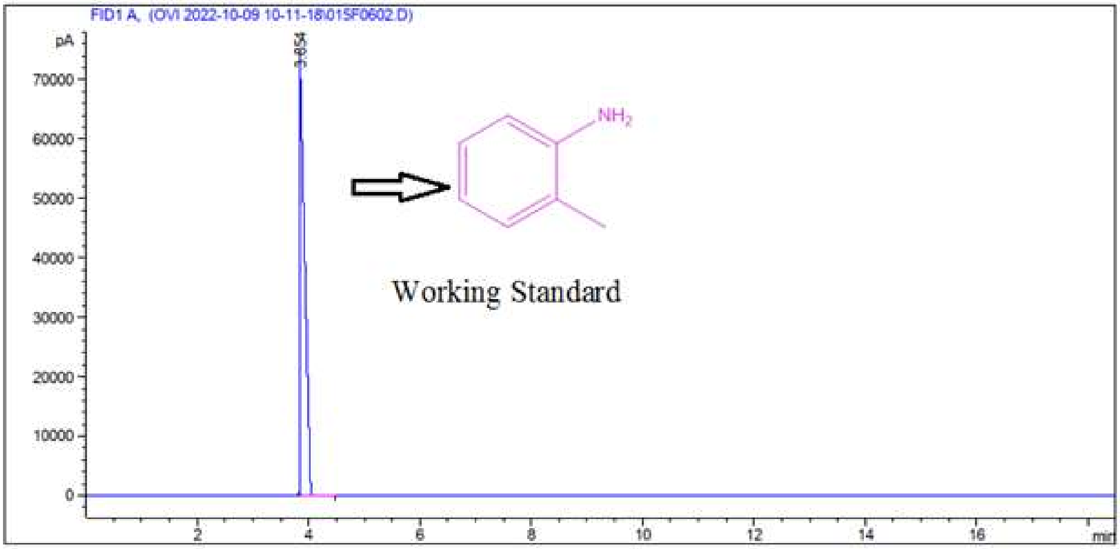


```
Signal 1: FID1 A,

Peak RetTime  Type   Width     Area      Area    Name
  #   [min]          [min]    [pA*s]      %
----|-------|------|-------|----------|--------|---------------------
   1   3.854 VB S    0.0984 4.39521e5          ?
```

**Catalyst**	**Substrate**	**Product**	**Catalyst ratio (mol%)**	**Retention time (RT) (min)**	**Area**	**Purity (%)**
**NA**	NA	o-toluidine	NA	3.854	439521	100

Figure S50**.** Chromatogram output for o-toluidine working standard

```
Additional Info : Peak(s) manually integrated
```

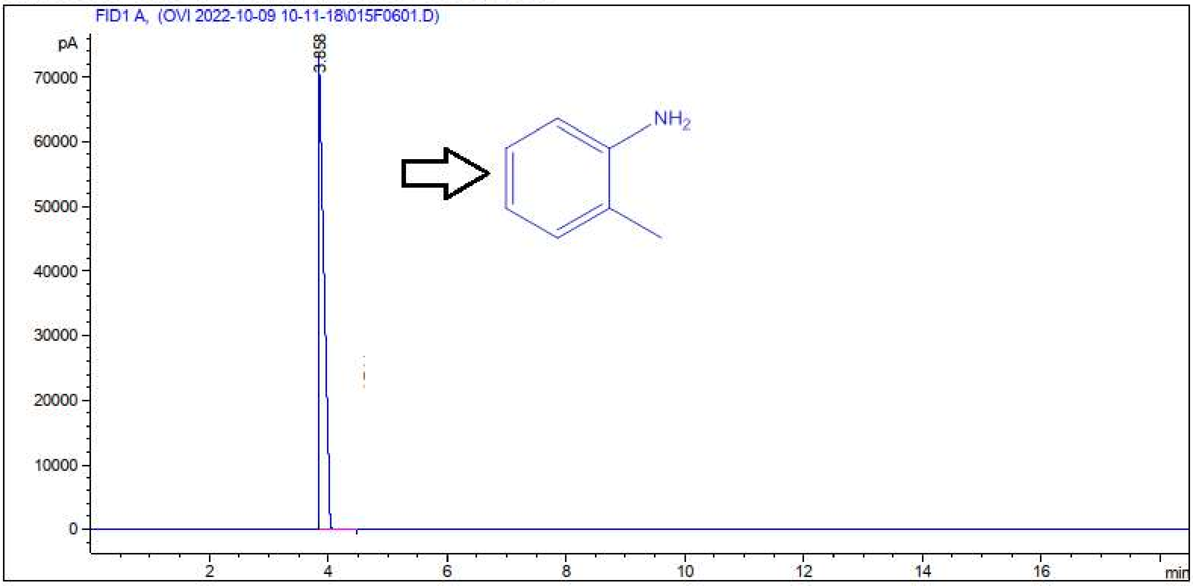


```
Signal 1: FID1 A,

Peak RetTime  Type   Width     Area      Area    Name
  #   [min]          [min]   [pA*s]       %
----|-------|------|-------|----------|--------|---------------------
   1   3.858 VB S    0.0971 4.06573e5
```

Catalyst	Substrate	Product	Catalyst ratio (mol%)	Retention time (RT) (min)	Area	Substrate conversion (%)
**Mixed MOF-Salinidol/Pd(II)**	1-methyl-2-nitrobenzene	o-toluidine	0.1	3.858	406573	92.5

Figure S51. Chromatogram output for o-toluidine result of hydrogenation reaction for nitrobenzene and derivatives
